# Evaluation of the cost-utility of phosphate binders as a treatment option for hyperphosphatemia in chronic kidney disease patients: a systematic review and meta-analysis of the economic evaluations

**DOI:** 10.1007/s10198-021-01275-3

**Published:** 2021-03-06

**Authors:** Kamolpat Chaiyakittisopon, Oraluck Pattanaprateep, Narisa Ruenroengbun, Tunlanut Sapankaew, Atiporn Ingsathit, Gareth J. Mckay, John Attia, Ammarin Thakkinstian

**Affiliations:** 1 Department of Clinical Epidemiology and Biostatistics, Faculty of Medicine, Ramathibodi Hospital, Mahidol University, 3rd Floor, Research Center Building, 270 RAMA VI Road. Ratchathewi, Bangkok, 10400 Thailand; 2 Department of Community Pharmacy, Faculty of Pharmacy, Silpakorn University, Nakhon Pathom, Thailand; 3 Department of Pharmacy, Faculty of Pharmacy, Silpakorn University, Nakhon Pathom, Thailand; 4 Center for Public Health, School of Medicine, Dentistry and Biomedical Sciences, Queen’s University Belfast, Belfast, UK; 5 Centre for Clinical Epidemiology and Biostatistics, School of Medicine and Public Health, Hunter Medical Research Institute, University of Newcastle, New Lambton, NSW Australia

**Keywords:** Economic evaluation, Hyperphosphatemia, Incremental net benefit, Meta-analysis, Phosphate binders, I10

## Abstract

**Background:**

Uncontrolled hyperphosphatemia in chronic kidney disease (CKD) patients commonly results in vascular calcification leading to increased risk of cardiovascular disease. Phosphate binders (PBs) are used for hyperphosphatemia and can be calcium-based (CBPBs) or non-calcium-based (NCBPBs), the latter being more expensive than CBPBs. In this study, we used meta-analysis approaches to assess the cost-utility of PBs for hyperphosphatemia in CKD patients.

**Methods:**

Relevant studies published prior to June 2019 were identified from PubMed, Scopus, the Cochrane Library, the National Health Service Economic Evaluation Database, and the Cost-Effectiveness Analysis Registry. Studies were eligible if they included CKD patients with hyperphosphatemia, compared any PBs and reported economic outcomes. Meta-analysis was applied to pool incremental net benefit (INB) across studies stratified by country income.

**Results:**

A total of 25 studies encompassing 32 comparisons were eligible. Lanthanum carbonate, a NCBPB, was a more cost-effective option than CBPBs in high-income countries (HICs), with a pooled INB of $3984.4 (599.5–7369.4), especially in pre-dialysis patients and used as a second-line option with INBs of $4860.2 (641.5–9078.8), $4011.0 (533.7–7488.3), respectively. Sevelamer, also a NCBPB, was not more cost-effective as a first-line option compared to CBPBs with a pooled INB of $6045.8 (− 23,453.0 to 35,522.6) and $34,168.9 (− 638.0 to 68,975.7) in HICs and upper middle-income countries, respectively.

**Conclusions:**

Lanthanum carbonate was significantly more cost-effective than CBPBs as a second-line option for hyperphosphatemia in pre-dialysis patients in HICs. However, the use of sevelamer is not more cost-effective as a first-line option compared to CBPBs.

**Supplementary Information:**

The online version contains supplementary material available at 10.1007/s10198-021-01275-3.

## Introduction

Chronic kidney disease (CKD) represents a significant *global public health burden* with high economic costs related to morbidity and mortality [1]. In 2016, global CKD prevalence was estimated to exceed 13%, of which four-fifths were classified as CKD stage 3 or higher [2]. Progression of *CKD* to end-stage renal disease (ESRD) increases the likelihood of complications particularly mineral and bone disorders, kidney failure and cardiovascular disease (CVD) [1, 3, 4].

Given the kidneys are responsible for the excretion of excess phosphorous, CKD leads to hyperphosphatemia in 40–85% [5–12]. Left untreated, the sequelae are renal osteodystrophy, secondary hyperparathyroidism, and vascular calcification [1, 11–13] leading to increased risk of CVD [14–17]. As such, clinical practice a few guidelines [13, 18, 19] recommend the prescription of phosphate binders (PBs), e.g., calcium-based PBs (CBPBs), as the initial management for hyperphosphatemia. If CBPBs are not effective, or there are contraindications for CBPBs, non-calcium-based PBs (NCBPBs, e.g., sevelamer, lanthanum carbonate, sucroferric oxyhydroxide or ferric citrate) are the next treatment options.

Previous systematic reviews (SR) and network meta-analyses (MA) [20–23] reported significantly increased mortality in CBPBs relative to NCBPBs [21]. CBPBs may increase hypercalcemia relative to NCBPBs, which may subsequently escalate the risk of CVD events [20, 22, 23]. These findings were consistent with those of conventional MA [24, 25] and observational studies [26, 27]. Consequently, clinical practice guidelines [13, 18, 19] have recommended NCBPBs instead of CBPBs. However, given NCBPBs are more expensive than CBPBs, they are not always regarded as the primary treatments due to affordability and availability [19].

Many economic evaluation (EE) studies and SRs assessed the cost-effectiveness of PBs [28, 29]. Studies conducted in high-income countries (HICs) suggested sevelamer and lanthanum carbonate may be more cost-effective treatments compared to CBPBs in patients with CKD. However, the cost-effectiveness of these treatments in low- and middle-income countries (LICs and MICs) has not been sufficient due to a lack of published data. Furthermore, evidence related to newer NCBPBs (e.g., ferric citrate, sucroferric oxyhydroxide, etc.) has not been previously reviewed.

Therefore, this SR and MA assessed the cost-effectiveness of PBs available under current practice guidelines for the treatment of hyperphosphatemia in CKD patients. Incremental net benefit (INB) for various PB comparisons were pooled and stratified by country income to provide reliable evidence for further consideration by policymakers.

## Methods

This SR and MA was performed in accordance with the Preferred Reporting Items for Systematic Reviews and Meta-Analyses Protocols (PRISMA-P) [30] and registered at PROSPERO (CRD42019145280).

### Data sources

A literature search up to June 2019 was performed in PubMed, Scopus, the Cochrane Central Register of Controlled Trials, the National Health Service Economic Evaluation Database (NHS EED), and the Cost-Effectiveness Analysis (CEA) Registry by Tufts Medical Center. Lists of references from selected articles/SRs were also checked to identify additional relevant studies. The search terms were constructed based on interventions/comparators (i.e., phosphate binder, calcium carbonate, sevelamer, lanthanum, sucroferric) and outcomes (i.e., economic evaluation, cost-utility analysis, and incremental net benefit), see more detail in Supplementary Methods S1.

### Study selection

Two reviewers (KC and NR) independently screened titles and abstracts, full articles were reviewed if a decision could not be made. Any disagreement was discussed with a third party (OP). EE studies (e.g., cost-utility analysis (CUA) or cost-effectiveness analysis (CEA)) were eligible if they met the following criteria: adult CKD with hyperphosphatemia, compared any pair of PBs regardless of dosage and treatment duration (CBPBs: calcium carbonate/acetate; NCBPBs: sevelamer, lanthanum carbonate, ferric citrate, sucroferric oxyhydroxide, aluminum hydroxide, colestimide, bixalomer, nicotinic acid), and any EE outcomes including incremental cost-effectiveness ratio (ICER), incremental cost-utility ratio (ICUR), INB/net monetary benefit (NMB), incremental cost (ΔC), incremental effectiveness (ΔE, e.g., life years (LYs) gained/lost and quality-adjusted life years (QALYs)). The following studies were ineligible: cost-minimization analysis and insufficient data for pooling despite three data requests to the corresponding author.

### Data extraction

Data extraction was performed independently by two of the three reviewers (KC, TS and NR). Any disagreement was discussed and resolved by a third party (OP). The data extraction forms (see Supplementary Methods S2) were designed by incorporating information from the consolidated health economic evaluation reporting standard (CHEERS) statement [31, 32], the NHS-EED [33], and the centre for reviews and dissemination guidance [34] consisting five parts: general information, study characteristics, participant characteristics and intervention/comparison, methods and outcomes of EE, and data for pooling.

The economic parameters including mean cost/ΔC, effectiveness/ΔE, and ICERs, along with dispersion (standard deviation (SD) or 95% confidence interval (CI)) were extracted. If not explicitly reported, they were extracted from cost-effectiveness plane graphs instead. Cost-effectiveness (C/E) threshold or willingness to pay (WTP) were also extracted, and if not reported, were based on the 2019 national agencies threshold (e.g., Canadian Agency for Drugs and Technologies in Health (CADTH), NICE) for that country.

### Risk of bias assessment

Risk of bias was assessed using the Bias in Economic Evaluation (ECOBIAS) checklist consisting of the general domain (11 items) and model-specific issues in economic evaluations including structure (4 items), data (6 items), and internal consistency (1 item), see Supplementary Methods S3 [35]. Every item was rated as yes, no, partly, unclear or not applicable.

### Interventions and economic outcomes

Interested interventions were comparisons of PBs including sevelamer versus CBPBs, lanthanum carbonate versus CBPBs, lanthanum carbonate versus sevelamer, and sucroferric oxyhydroxide versus sevelamer. The economic outcome measure was INB [36–38], which was calculated as follows: INB = K(ΔE)−ΔC, where K is the C/E threshold or WTP, ΔE, and ΔC are the difference of QALYs and cost between intervention and comparator. Those studies reported the ICERs were converted to the INB as INB = ΔE (K−ICER). However, variation in data reporting of EE studies necessitated the estimation of INB and variance based on five scenarios in line with previous recommendations (Supplementary Methods S4A) [39, 40]. The intervention was characterized as cost-effective if the INB was positive (i.e., favoring the intervention), otherwise the new intervention was characterized as not cost-effective [38, 41].

### Currency conversion

Individual studies used various currencies and year by country. The currency was converted to a 2019 cost metric using the consumer price index (CPI) [42] and United States dollar (*US*$) using purchasing power parity (PPP) [43], see Supplementary Methods S4B.

### Statistical analysis

Pairwise MA of pooled INB stratified by country income was applied for each PB comparison if there were at least 2 EE studies [44] (i.e., HICs, upper-middle-income countries (UMICs), lower-middle-income countries (LMICs), and LICs). A fixed-effect model using the inverse-variance method was used if heterogeneity was not present, otherwise a random effect model (DerSimonian and Laird) was applied, see Supplementary Methods S4C.

Heterogeneity was assessed by the Cochrane’s *Q* test and *I*^*2*^ statistic; a *Q* test *p* value < 0.1 or an *I*^*2*^ value > 25% was regarded as significant. Sources of heterogeneity were explored by fitting each co-variable independently within a meta-regression model e.g., pre-dialysis versus dialysis, first- versus second-line treatment, C/E thresholds, lifetime versus non-lifetime horizon (defined as the duration of study over which costs and outcomes are calculated), model discount rate (defined as the rate for adjusting future costs and outcomes to the present value in the economic model). A co-variable was considered a source of heterogeneity if the decrease in Tau^2^ ≥ 50%. Subgroup analysis was performed accordingly. In addition, a 95% prediction interval (PI) was estimated where there were at least three studies to predict if the pooled INB was still cost-effective in other settings [45, 46].

Publication bias was assessed using a funnel plot and Egger’s test. Indications of asymmetry were addressed through the use of contour enhanced funnel plots to distinguish the source of asymmetry. All data pooling was undertaken using Microsoft® excel version 2019 and analysed by STATA® version 16. A two-sided *p* value < 0.05 was considered statistically significant.

## Results

### Study identification

**Fig. 1:**
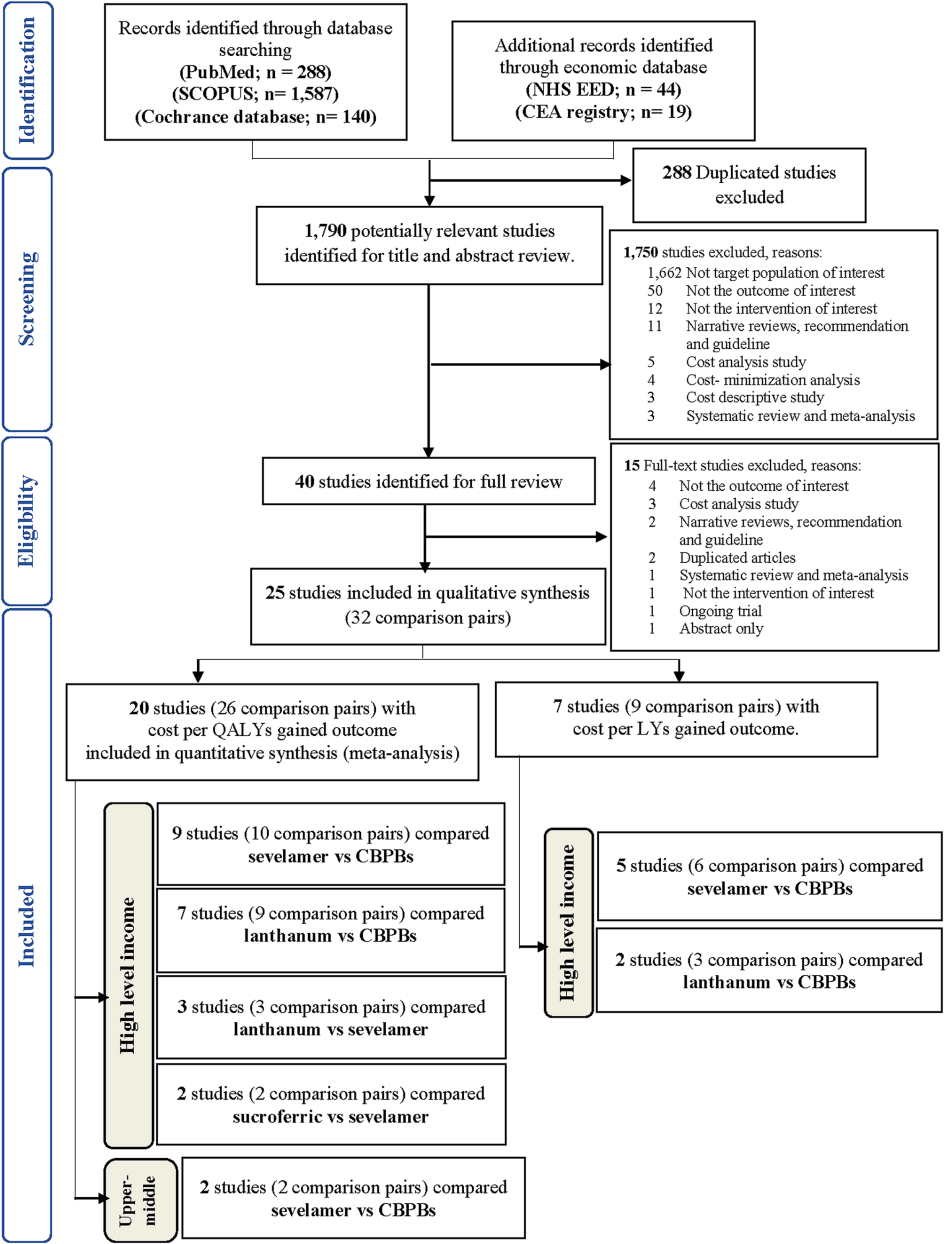
PRISMA flow diagram

A total of 1790 studies were identified, of which 25 studies were eligible, comprising 32 comparisons [47–71] (Fig. 1). Among them, 20 studies [47–66] (number comparisons (*n*) = 26) and 7 studies [59, 60, 67–71] (*n* = 9) provided data on CUA in cost/QALYs and CEA in cost/LYs, respectively. Of the CUA studies, 18 [49–66] and 2 [47, 48] were conducted in HICs and UMICs, respectively. Four comparisons of PBs were included: sevelamer versus CBPBs (*N* = 9 for HICs [49–57] and *N* = 2 for UMICs [47, 48]), lanthanum carbonate versus CBPBs (*N* = 7 for HICs [49, 57–62]), lanthanum carbonate versus sevelamer (*N* = 3 for HICs [49, 63, 64]), and sucroferric oxyhydroxide versus sevelamer (*N* = 2 for HICs [65, 66]). All CEA studies were from HICs with sevelamer versus CBPBs (*N* = 5 [67–71]) and lanthanum carbonate versus CBPBs (*N* = 2 [59, 60]). Only CUA studies were considered in MA for pooling INBs.

Study characteristics are reported (Table 1) and all applied the third-party payer’s perspective. Most studies used Markov models (*N* = 18 [47, 48, 50–60, 63–67]) and were evaluated over a patient’s lifetime (*N* = 21 [47–49, 51–62, 64–69]). Nineteen [48–53, 60–70] and eight studies [47, 54–59, 71] focused on dialysis and pre-dialysis patients, respectively. The majority of studies accounted for dialysis costs in their analyses, although 9 studies failed to do so [48–50, 63–66, 68, 69]. All studies, with the exception of two [70, 71], reported direct costs with discounting for both costs and outcomes. Sources for model input parameters (*N* = 14 [50, 51, 55, 58–66, 68, 69]) and utility data (*N* = 11 [47, 50, 51, 56, 60–66]) were mostly from multiple studies. The majority of studies used country-specific thresholds (*N* = 22 [49–52, 54–71]).

**Table 1:** Characteristics of included studies

Study	Country	EEs	Analytic approach	Target patient	Intervention/Comparator	Option	Outcome measure	Time horizon	Current year	Dialysis cost	Source of clinical	Source of utility	Discount rate	Threshold	Result
High-income country															
Huybrechts [68], 2005	US	CEA	DES	DD	Sevelamer/CBPBs	1st line	Lys	Lifetime	2002	No	Multiple studies	NA	3	$50,000	CE
Brennan [62], 2007	UK	CUA	Clinical pathway	DD	Lanthanum/CBPBs	2nd line	QALYs	Lifetime	2006	Yes	Multiple studies	Multiple studies	3.5	€ 30,000	CE
Manns [51], 2007	Canada	CUA	Markov	DD	Sevelamer/CBPBs	1st line	QALYs	Lifetime	2004	Yes	Multiple studies	Multiple studies	5	CAD$	Not CE
														75,000	
Taylor [50], 2008	UK	CUA	Markov	DD	Sevelamer/CBPBs	1st line	QALYs	5 year	2007	No	Multiple studies	Multiple studies	3	€ 30,000	CE
Huybrechts [69], 2009	Canada	CEA	DES	DD	Sevelamer/CBPBs	1st line	Lys	Lifetime	2005	No	Multiple studies	NA	3	CAD$	CE
														60,000	
Goto [61], 2011	Japan	CUA	Patient-level state	DD	Lanthanum/CBPBs	2nd line	QALYs	Lifetime	2010	Yes	Multiple studies	Multiple studies	3	¥ 50,00,000	CE
Park [63], 2011	US	CUA	Markov	DD	Lanthanum/Sevelamer	2nd line	QALYs	10 year	2009	No	Multiple studies	Multiple studies	5	$ 50,000	CE
Vegter (a) [59], 2011	UK	CUA	Markov	NDD	Lanthanum/CBPBs	2nd line	QALYs, Lys	Lifetime	2009	Yes	Multiple studies	Synthesis	3.5	€ 30,000	Domi-nant
		CEA													
Vegter (b) [59], 2011	UK	CUA	Markov	DD	Lanthanum/CBPBs	2nd line	QALYs, Lys	Lifetime	2009	Yes	Multiple studies	Synthesis	3.5	€ 30,000	CE
		CEA													
Vegter [60], 2012	Canada	CUA	Markov	DD	Lanthanum/CBPBs	2nd line	QALYs, Lys	Lifetime	2010	Yes	Multiple studies	Multiple studies	5	CAD$	CE
		CEA												50,000	
Bernard [52], 2013	UK	CUA	Markov	DD	Sevelamer/CBPBs	1st line	QALYs	Lifetime	2009	Yes	Single study	Synthesis	3.5	€ 30,000	CE
NICE (a) [49], 2013	UK	CUA	DES	DD	Sevelamer/CBPBs	1st line	QALYs	Lifetime	2012	No	Synthesis	Synthesis	3.5	€ 30,000	Not CE
NICE (b) [49], 2013	UK	CUA	DES	DD	Lanthanum/CBPBs	1st line	QALYs	Lifetime	2012	No	Synthesis	Synthesis	3.5	€ 30,000	Not CE
NICE (c) [49], 2013	UK	CUA	DES	DD	Lanthanum/Sevelamer	1st line	QALYs	Lifetime	2012	No	Synthesis	Synthesis	3.5	€ 30,000	CE
Thompson [54], 2013	UK	CUA	Markov	NDD	Sevelamer/CBPBs	1st line	QALYs	Lifetime	2011	Yes	Single study	Synthesis	3.5	€ 30,000	CE
Ruggeri [70], 2014	Italy	CEA	Trial-based	DD	Sevelamer/CBPBs	1st line	Lys	3 year	2012	Yes	Elicited in the study	NA	NA	€ 20,000	CE
Ruggeri (a) [71], 2015	Italy	CEA	Trial-based	NDD	Sevelamer/CBPBs	1st line	Lys	3 year	2012	Yes	Elicited in the study	NA	NA	€ 20,000	CE
Ruggeri (b) [71], 2015	Italy	CEA	Trial-based	NDD	Sevelamer/CBPBs	1st line	Lys	3 year	2012	No	Elicited in the study	NA	NA	€ 20,000	CE
Gonzalez-Parra [64], 2015	Spain	CUA	Markov	DD	Lanthanum/Sevelamer	1st line	QALYs	Lifetime	2012	No	Multiple studies	Multiple studies	3	€ 30,000	CE
Gros [58], 2015	Spain	CUA	Markov	NDD	Lanthanum/CBPBs	2nd line	QALYs	Lifetime	2013	Yes	Multiple studies	Synthesis	3	€ 30,000	Dominant
Gutzwiller [65], 2015	UK	CUA	Markov	DD	Sucroferric oxyhydroxide/Sevelamer	2nd line	QALYs	Lifetime	2012	No	Multiple studies	Multiple study	3.5	€ 20,000	Not CE
Panichi [67], 2015	Italy	CEA	Markov	DD	Sevelamer/CBPBs	1st line	Lys	Lifetime	2014	Yes	Elicited in the study	NA	NA	€ 30,000	CE
Del Pino [56], 2016	Spain	CUA	Markov	NDD	Sevelamer/CBPBs	1st line	QALYs	Lifetime	2014	Yes	Single study	Multiple studies	3	€ 37,500	CE
Nguyen [55], 2016	Singapore	CUA	Markov	NDD	Sevelamer/CBPBs	1st line	QALYs	Lifetime	2013	Yes	Multiple studies	Single studies	3.5	S$ 61,000	CE
Cho [53], 2017^a^	Korea	CUA	Markov	DD	Sevelamer/CBPBs	1st line	QALYs	Lifetime	2015	Yes	Single study	Synthesis	5	₩ 3,18,94,720	CE
Habbous (a) [57], 2017	Canada	CUA	Markov	NDD	Sevelamer/CBPBs	1st line	QALYs	Lifetime	2015	Yes	Synthesis	Synthesis	1.5	CAD$	not CE
														60,000	
Habbous (b) [57], 2017	Canada	CUA	Markov	NDD	Lanthanum/CBPBs	1st line	QALYs	Lifetime	2015	Yes	Synthesis	Synthesis	1.5	CAD$	Not CE
														60,000	
Habbous (c) [57], 2017	Canada	CUA	Markov	DD	Sevelamer/CBPBs	1st line	QALYs	Lifetime	2015	Yes	Synthesis	Synthesis	1.5	CAD$	Not CE
														60,000	
Habbous (d) [57], 2017	Canada	CUA	Markov	DD	Lanthanum/CBPBs	1st line	QALYs	Lifetime	2015	Yes	Synthesis	Synthesis	1.5	CAD$	Not CE
														60,000	
CADTH [66], 2019	Canada	CUA	Markov	DD	Sucroferric oxyhydroxide/Sevelamer	2nd line	QALYs	Lifetime	2018	No	Multiple study	Multiple studies	1.5	CAD$	CE
														60,000	
Upper-middle income country															
Yang [48], 2016^a^	China	CUA	Markov	DD	Sevelamer/CBPBs	1st line	QALYs	Lifetime	2015	No	Single study	Single study	3.5	CNY 1,51,070	CE
Goh [47], 2018^a^	Malaysia	CUA	Markov	NDD	Sevelamer/CBPBs	1st line	QALYs	Lifetime	2013	Yes	Single study	Multiple studies	3	RM 66,264	CE

*CAD$* Canadian dollar, *CBPBs* calcium-based phosphate binders, *CEA* cost-effectiveness analysis, *CNY* *Chinese* Yuan, *CUA* cost-utility analysis, *DD* dialysis patient, *DES* Discrete event simulation, *EEs* economic evaluations, *€* euro, *LYs* life years, *NA* not applicable, *NDD* non-dialysis or pre-dialysis patient, *QALYs* Quality-adjusted life years, *RM* Ringgit *Malaysia*, *S$* *Singapore* dollar, *UK* United Kingdom, *US* United States, *₩* won, *¥* Yen

^a^Gross domestic product (GDP) based threshold

Twenty-one of the 25 studies reported that NCBPBs were cost-effective, of which 19 [49, 50, 52–56, 58–62, 64, 66–71] and two studies [47, 48] originated from HICs and UMICs, respectively. For the 19 studies from HICs, 11 [50, 52–56, 67–71] concluded that sevelamer was more cost-effective than CBPBs. Five [58–62] and two studies [49, 64] found lanthanum carbonate was more cost-effective than CBPBs and sevelamer, respectively. And a single study reported that sucroferric oxyhydroxide was more cost-effective than sevelamer [66]. For UMICs, both studies reported that sevelamer was more cost-effective than CBPBs [47, 48].

### Risk of bias assessment

Results from a risk of bias assessment are described, see Supplementary Table S1. For overall bias, all studies were regarded as unbiased on perspective, comparator, ordinal ICER, and reporting/dissemination. Between 84–96% of studies were unbiased on data collection, valuation, double counting and discounting. Seven studies demonstrated partial bias using one-way sensitivity analysis [49, 50, 52, 55, 56, 60, 62]. All studies were regarded as unbiased on structural assumptions, treatment comparator, utility weights, and on internal consistency of model-specific bias. Several studies demonstrated partial bias on the basis of time horizon [50, 70], data identification [50, 67], non-transparent data [60, 62] and baseline data [67]. A single study provided insufficient detail for assessment on wrong model bias [62].

### Sevelamer versus CBPBs

**Fig. 2:**
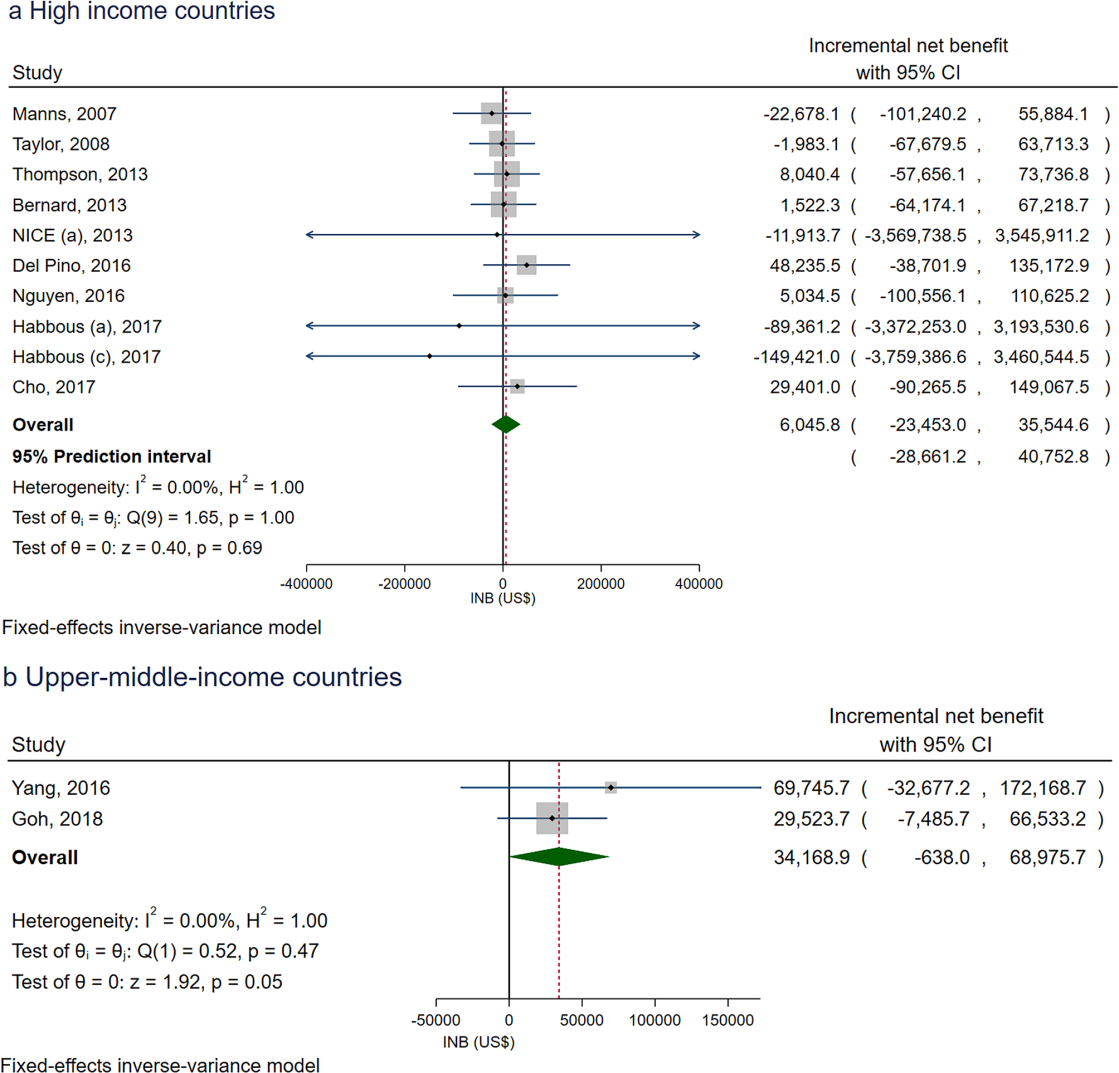
Overall pooling of the incremental net benefits of sevelamer versus calcium-based phosphate binders in **a** high-income countries and **b** upper-middle income countries

Twelve comparisons (*N* = 11 [47–57]) between sevelamer and CBPBs were CUAs, with 10 and two in HICs [49–57] and UMICs [47, 48] (Supplementary Table S2). All studies evaluated PBs as primary interventions. Among HICs, INBs were homogenous (*I*^*2*^ = 0%) representing a pooled INB (95% CI) of $6045.8 (− 23,453.0 to 35,522.6), see Fig. 2a, suggesting sevelamer was more cost-effective than CBPBs, although this failed to reach statistical significance. The 95% PI also suggested that the true effect in future setting could be null or in the similar direction of the pooled INB, with the range of − $28,661.2 to $40,752.8.

In addition, subgroup analysis was undertaken according to pre-dialysis/dialysis patients with and without consideration of dialysis cost, and a median C/E threshold (< $48,114.6 versus ≥ $48,114.6), see Supplementary Figure S1. Sevelamer was more cost-effective than CBPBs in pre-dialysis, but not in dialysis patients, with and without accounting for dialysis costs (i.e., pooled INBs were $19,145.8 (− 27,797.5 to 66,089.2), − $1.986.5 (− 67.671.7 to 63,698.8), and − $2760.8 (− 49,203.4 to 43,681.8), respectively, but again all estimates failed to reach significance. Likewise, subgroup analysis based on the median threshold value of $48,114.6 failed to identify significant differences in associated costs. The 95%PIs according to subgroup analysis included the null effect, which were consistent with the pooled INBs.

All studies, bar one [50], were evaluated over a patient’s lifetime horizon. Sensitivity analysis was performed by excluding the study that used non-lifetime horizon [50] and the highest threshold studies [55], providing pooled INBs of $8073.3 (− 24,940.7 to 41,087.4), and $6131.4 (− 24,590.7 to 36,853.5) respectively (Supplementary Figure S2), which were consistent with the overall pooled INBs.

For UMICs, INBs of sevelamer versus CBPBs were pooled across two studies [47, 48] with values of $34,168.9 (− 638.0 to 68,975.7), suggesting sevelamer was more cost-effective than CBPBs, although this was also not significant, see Fig. 2b. The funnel plot and Egger’s test did not identify any asymmetry for pooling INBs in HICs and UMICs (see Supplementary Figure S3 and Table S3).

### Lanthanum carbonate versus CBPBs

**Fig. 3:**
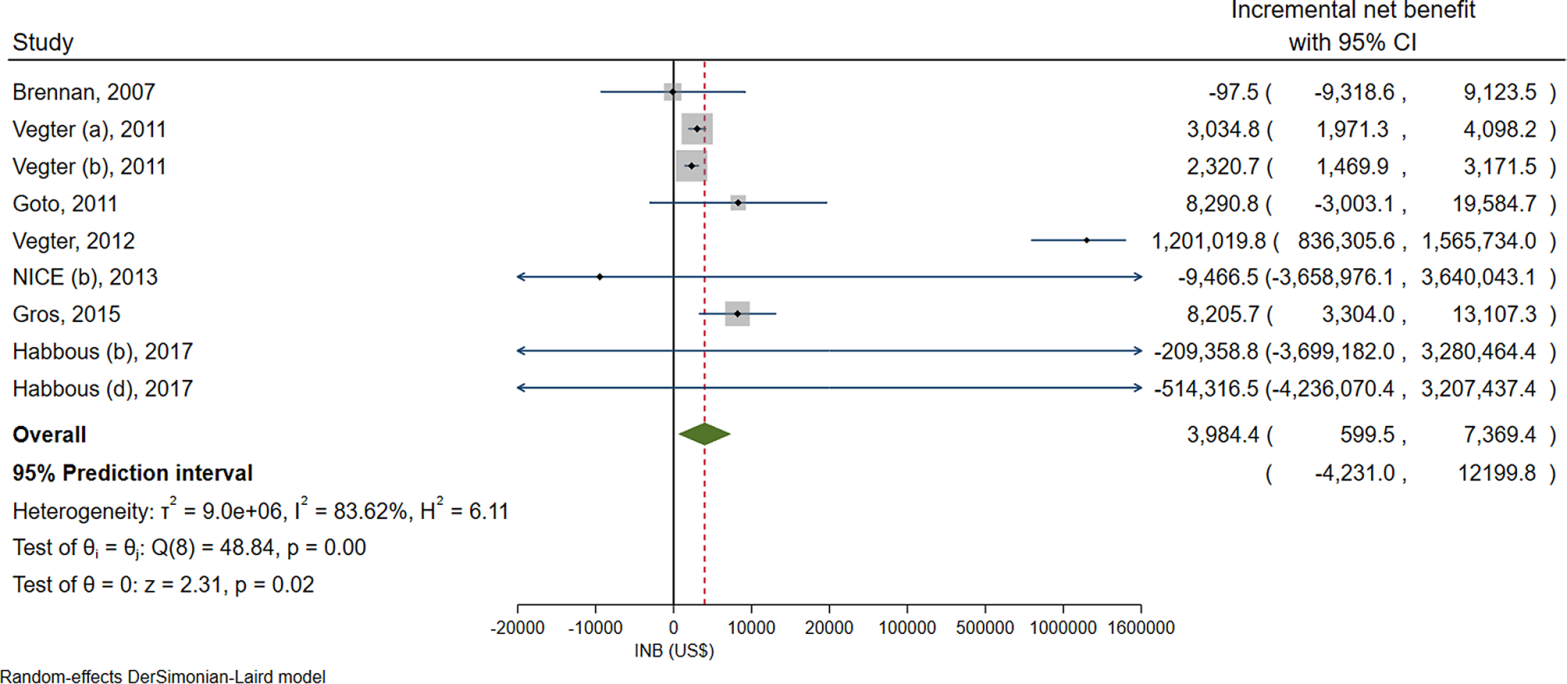
Overall pooling of the incremental net benefits of lanthanum carbonate versus calcium-based phosphate binders in high-income countries

Nine comparisons (*N* = 7 [49, 57–62]) evaluated CUAs between lanthanum carbonate and CBPBs in HICs. Three [49, 57] and six [58–62] focused on first and second-line treatments respectively, see Supplementary Table S2. High heterogeneity was observed (*I*^*2*^ = 83.6%) with a pooled INB of $3984.4 (599.5–7369.4) indicating lanthanum carbonate was significantly more cost-effective than CBPBs, see Fig. 3. However, the 95% PI was − $4231.0 to $12,199.8 indicating lanthanum carbonate was not cost-effective than CBPBs in other settings.

The source of heterogeneity was explored by considering each co-variable in a meta-regression model one by one including pre-dialysis versus dialysis, first- versus second-line treatment, discount rates, and C/E thresholds. None of them could reduce heterogeneity but worsen in increasing heterogeneity with the *I*^*2*^ for these corresponding co-variables of 85.1, 85.7, 84.1, and 84.1%, see Supplementary Table S4. A subgroup analysis suggested lanthanum carbonate was significantly more cost-effective relative to CBPBs in pre-dialysis patients, second-line treatment, discount rate < 3.5%, and at a median threshold ≥ $45,645.8 with pooled INBs of $4860.2 (641.5–9078.8), $4011.0 (533.7–7488.3), $8218.0 (3721.6–12,714.4), and $8218.0 (3721.6–12,714.4), respectively. However, none of these 95% PIs was statistically significant, see Supplementary Figure S4.

All studies, except one [49], considered dialysis costs in their evaluation. Sensitivity analyses excluding this study [49], and other studies with the highest discount rate [60] and C/E threshold [61] provided pooled INBs of $3660.1 (132.3–7187.8), $2761.0 (1996.0–3526.1), and $3993.6 (576.5–7410.7), respectively (Supplementary Figure S5). These were consistent with the overall pooled INB. Moreover, the exclusion of the highest discount rate study [60] reduced the *I*^*2*^ statistic for heterogeneity significantly from 83.6 to 5.1%, and its 95% PI of $1573.5–$3948.6 was still statistically significant.

The Egger’s test and the funnel plots suggested asymmetry for pooling of INBs (see Supplementary Table S3 and Figure S6). A contour enhanced funnel plot placed most of the studies in the non-significant area suggesting asymmetry may be due both to heterogeneity or missing positive studies (see Supplementary Figure S6).

### Lanthanum carbonate versus sevelamer

**Fig. 4:**
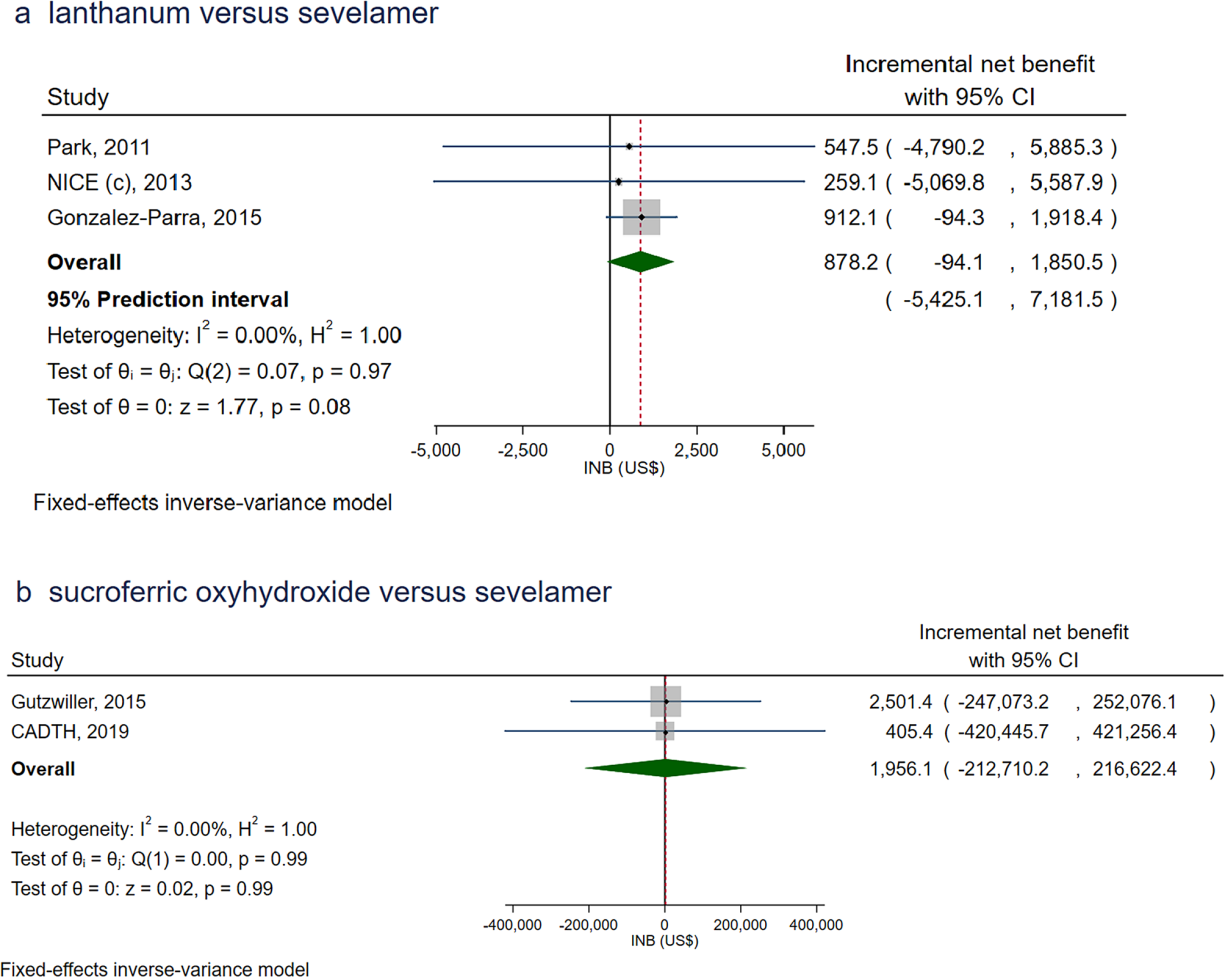
Overall pooling of the incremental net benefits of **a** lanthanum carbonate versus sevelamer and **b** sucroferric oxyhydroxide vs sevelamer, in high-income countries

Three studies [49, 63, 64] compared CUAs between lanthanum carbonate and sevelamer and all focused on dialysis patients and were conducted in HICs (see Supplementary Table S2). Two studies [49, 64] evaluated first-line PB treatments and a single study [63] evaluated second-line PB treatments. There was no heterogeneity (*I*^*2*^ = 0%) with the pooled INBs of $878.2 (− 94.1 to 1850.5), indicating lanthanum carbonate was more cost-effective than sevelamer, although this failed to reach significance, see Fig. 4a. The 95% PI also suggested that the true effect in future setting could be null or in the similar direction of the pooled INB, with the range of − $5425.1 to $7181.5. Neither the Egger’s test (see Supplementary Table S3) nor the funnel plot (see Supplementary Figure S7) indicated asymmetry or publication bias.

### Sucroferric oxyhydroxide versus sevelamer

Only two studies [65, 66] compared CUAs of sucroferric oxyhydroxide to sevelamer, and these were based in HICs (see Supplementary Table S2) and evaluated PBs as second-line treatments in dialysis patients without considering dialysis costs. The pooled INB was $1956.1 (− 212,710.2 to 216,622.4) with no heterogeneity (*I*^*2*^ = 0%); the very wide confidence intervals, limited any inference, see Fig. 4b. Neither the Egger’s test (see Supplementary Table S3) nor the funnel plot (see Supplementary Figure S8) were asymmetrical indicating no publication bias.

## Discussion

We conducted SR and MA for the economic evaluation of PBs for hyperphosphatemia treatments in CKD patients stratified by country income. Our findings identified lanthanum carbonate as a significantly more cost-effective second-line treatment in HICs compared to CBPBs, especially in pre-dialysis patients and in countries with a C/E threshold value ≥ $45,645.8. There was the suggestion of improved cost-effectiveness of sevelamer as a first-line treatment compared to CBPBs in both HICs and UMICs, but this was not significant. In addition, both lanthanum carbonate and sucroferric oxyhydroxide were more cost-effective than sevelamer, but these were also not significant.

Previous SRs of EE have provided limited qualitative comparisons of PBs without MAs [28, 29]. The evidence to date supports the cost-effective use of NCBPBs and lanthanum carbonate in particular. This evidence may guide clinical and safety considerations as NCBPBs have been reported in lowering side effects, hypercalcemia, CVD events and mortality compared to CBPBs [20–23]. The economic benefits and reduced expenditure associated with NCBPB usage, coupled with reduced side effects and complications, lead to improved patient adherence and quality of life. Our findings will better inform the drug selection process for clinicians, researchers, and policymakers. The first-line treatment options favor sevelamer or CBPBs as the drug of choice in both HICs and UMICs, although this doesn’t reach statistical significance. Lanthanum carbonate may offer a better second-line treatment option than CBPBs especially in pre-dialysis patients and in countries with a 2019 C/E threshold in excess of $45,645.8. Nevertheless, budget impact analysis will be necessary to assess affordability in each country’s drug selection process.

Our study had several strengths. We applied MA for estimate overall INBs to determine the most cost-effective treatment options. INBs were more amenable to pooling than ICERs which are more difficult to interpret [72–74]. The interested treatment was determined as more cost-effective if the INB was positive, representing a simple and uncomplicated message for the benefit of clinicians, researchers and policymakers [73]. We extracted data from individual EE studies, which reported results under five scenarios accounting for the type of patients, treatment strategies, clinical/cost/humanistic data, perspective, time horizon, and C/E thresholds [39, 40]. In addition, accurate comparisons of the EE studies required currency conversions standardized to US dollars using 2019 CPI and PPP conversions. Furthermore, stratified analysis by country income offered improved sensitivity given the variation in healthcare provision and service delivery systems. Lastly, subgroup and sensitivity analyses were performed according to test the robustness of the findings reported.

Our study also had several limitations. Firstly, the majority of studies were conducted in HICs limiting the generalizability. Secondly, the pooled INB comparison of lanthanum carbonate to CBPBs, which was the only statistically significant result, was subject to a high level of heterogeneity, although the sensitivity analysis significantly reduced the level of heterogeneity and potential bias observed. Thirdly, the pooled INBs of lanthanum carbonate and sucroferric oxyhydroxide for comparison to sevelamer were based on a small number of studies, limiting the robustness of the findings reported and also the assessment of publication bias. Finally, no studies were identified that evaluated the cost-effectiveness of sevelamer as a second-line treatment for comparison to CBPBs, which requires further consideration. Further EE studies are necessary to extend these results to UMICs, MICs, and LICs, as well as studies to evaluate the cost-effectiveness of NCBPBs as first- and second-line treatment options in both pre-dialysis and dialysis patients. In addition, a network meta-analysis should be further applied to compare cost-effectiveness of all possible treatment regimens.

## Conclusion

Our data identified lanthanum carbonate might be more cost-effective as a second-line treatment for hyperphosphatemia in pre-dialysis patients than CBPBs in HICs. The use of sevelamer as a first-line treatment may also offer some savings over CBPBs in HICs and UMICs. The inclusion of additional studies as they become available, especially from UMICs, MICs, and LICs, will inform improved cost-effectiveness for the hyperphosphatemia treatments in different healthcare settings.

## Supplementary Information

Below is the link to the electronic supplementary material.

Supplementary file1 (DOCX 22126 KB)
