# Supplementary material for: Evaluation of the cost-utility of phosphate binders as a treatment option for hyperphosphatemia in chronic kidney disease patients: a systematic review and meta-analysis of the economic evaluations
Source: Eur J Health Econ. 2021 Mar 6;22(4):571–84. doi: 10.1007/s10198-021-01275-3 (PMC8166732; doi:10.1007/s10198-021-01275-3)
Supplement: Supplementary file 1 — Supplementary file1 (DOCX 22126 KB) [file 10198_2021_1275_MOESM1_ESM.docx]

**Supplementary materials**

**Evaluation of the cost utility of phosphate binders as a treatment option for hyperphosphatemia in chronic kidney disease patients:**

**A systematic review and meta-analysis of the economic evaluations**

# Supplement legends

| **Methods S1.** | Search terms and strategy | *Page 2* |
| --- | --- | --- |
| **Methods S2.** | Data extraction form | *Page 6* |
| **Methods S3.** | ECOBIAS checklist | *Page 13* |
| **Methods S4.** | Data preparation and statistical analysis | *Page 14* |
| **Table S1.** | Risk of bias assessment based on ECOBIAS checklist | *Page 17* |
| **Table S2.** | Description of the incremental cost-effectiveness ratio (ICER) and incremental net benefit (INB) of included studies stratified by level of country income | *Page 19* |
| **Table S3.** | Publication bias assessments for meta-analysis | *Page 20* |
| **Table S4.** | Evaluation of heterogeneity of INB for lanthanum carbonate versus calcium-based phosphate binders (CBPBs) through covariable meta-regression adjustment | *Page 21* |
| **Figure S1.** | Subgroup analysis of pooled INBs for sevelamer versus CBPBs in high-income countries by a) patients with or without consideration of dialysis cost, and b) median cost-effectiveness (C/E) threshold. | *Page 22* |
| **Figure S2.** | Sensitivity analysis of pooled INBs for sevelamer versus CBPBs in high-income countries through study omission | *Page 23* |
| **Figure S3.** | Funnel plots for INBs of sevelamer versus CBPBs in high- and upper-middle income countries. | *Page 24* |
| **Figure S4.** | Subgroup analysis of pooled INBs for lanthanum carbonate versus CBPBs in high-income countries by a) pre-dialysis and dialysis patients, b) treatment, c) discount rate, and d) median C/E threshold. | *Page 25* |
| **Figure S5.** | Sensitivity analysis of pooled INBs for lanthanum carbonate versus CBPBs in high-income countries through study omission | *Page 27* |
| **Figure S6.** | Funnel plot and contour-enhanced funnel plot for INBs of lanthanum carbonate versus CBPBs in high-income countries**.** | *Page 28* |
| **Figure S7.** | Funnel plot for INBs of lanthanum carbonate versus sevelamer | *Page 29* |
| **Figure S8.** | Funnel plot for INBs of sucroferric oxyhydroxide versus sevelamer | *Page 30* |
| **Reference** | Reference of supplementary materials | *Page 31* |

**Methods S1. Search terms and strategy**

**Search strategy in MEDLINE via PubMed.**

| **Domain(s)** | **Search** | **Query** |
| --- | --- | --- |
| **Intervention/ Comparison (I/C):**  Among phosphate binders | #1  #2  #3  #4  #5  #6  #7  #8  #9  #10  #11  #12  #13  #14  #15 | “phosphate bind*”  “phosphate lowering”  “calcium acetate”  “calcium carbonate”  “lanthanum”  “sevelamer”  “ferric citrate”  “sucroferric”  “aluminum hydroxide”  “colestimide”  “cholebine”  “bixalomer”  “nicotinic acid”  “niacin”  “vitamin B3” |
|  | #16 | Search #1 OR #2 OR #3 OR #4 OR #5 OR #6 OR #7 OR #8 OR #9 OR #10 OR #11 OR #12 OR #13 OR #14 OR #15 |
| **Outcome (O):**  Economic outcomes | #17  #18  #19  #20  #21  #22  #23  #24  #25  #26  #27  #28  #29  #30  #31  #32  #33  #34  #35  #36  #37  #38  #39 | “Cost-Benefit Analysis"[Mesh]  "economic analysis"  "economic evaluation"  “cost effectiveness*”  “cost utility*”  “cost benefit*”  “ICER”  “ICUR”  "Incremental net benefit"  "INB"  "Net monetary benefit"  "NMB"  “incremental cost”  “delta cost”  “incremental effectiveness”  “incremental efficacy”  “delta effectiveness”  "Disability adjusted life years"  "DALY"  "Quality adjusted life years"  "QALY"  “monte carlo*”  "probabilistic sensitivity analysis" |
|  | #40 | Search #17 OR #18 OR #19 OR #20 OR #21 OR #22 OR #23 OR #24 OR #25 OR #26 OR # 27 OR #28 OR #29 OR #30 OR #31 OR #32 OR #33 OR #34 OR #35 OR #36 OR #37 OR #38 OR #39 |
| **I/C AND O** | #41 | Search #16 AND #40 |

**Search strategy in Scopus.**

| **Domain(s)** | **Search** | **Query** |
| --- | --- | --- |
| **Intervention/ Comparison (I/C):**  Among phosphate binders | #1  #2  #3  #4  #5  #6  #7  #8  #9  #10  #11  #12  #13  #14  #15 | “phosphate bind*”  “phosphate lowering”  “calcium acetate”  “calcium carbonate”  “lanthanum”  “sevelamer”  “ferric citrate”  “sucroferric”  “aluminum hydroxide”  “colestimide”  “cholebine”  “bixalomer”  “nicotinic acid”  “niacin”  “vitamin B3” |
|  | #16 | Search #1 OR #2 OR #3 OR #4 OR #5 OR #6 OR #7 OR #8 OR #9 OR #10 OR #11 OR #12 OR #13 OR #14 OR #15 |
| **Outcome (O):**  Economic outcomes | #17  #18  #19  #20  #21  #22  #23  #24  #25  #26  #27  #28  #29  #30  #31  #32  #33  #34  #35  #36  #37  #38  #39 | "economic analysis"  "economic evaluation"  “cost effectiveness*”  “cost utility*”  “cost benefit*”  “ICER”  “ICUR”  "Incremental net benefit"  "INB"  "Net monetary benefit"  "NMB"  “incremental cost”  “delta cost”  “incremental effectiveness”  “incremental efficacy”  “delta effectiveness”  “delta efficacy”  "Disability adjusted life years"  “DALY”  "Quality adjusted life years"  “QALY”  “monte carlo*”  "probabilistic sensitivity analysis" |
|  | #40 | Search #17 OR #18 OR #19 OR #20 OR #21 OR #22 OR #23 OR #24 OR #25 OR #26 OR # 27 OR #28 OR #29 OR #30 OR #31 OR #32 OR #33 OR #34 OR #35 OR #36 OR #37 OR #38 OR #39 |
| **I/C AND O** | #41 | Search #16 AND #40 |

**Search strategy in Cochrane database.**

| **Domain(s)** | **Search** | **Query** |
| --- | --- | --- |
| **Intervention/ Comparison:**  Among phosphate binders | #1  #2  #3  #4  #5  #6  #7  #8  #9  #10  #11  #12  #13  #14  #15 | phosphate bind*  “phosphate lowering”  “calcium acetate”  “calcium carbonate”  “lanthanum”  “sevelamer”  “ferric citrate”  “sucroferric”  “aluminum hydroxide”  “colestimide”  “cholebine”  “bixalomer”  “nicotinic acid”  “niacin”  “vitamin B3” |
|  | #16 | Search #1 OR #2 OR #3 OR #4 OR #5 OR #6 OR #7 OR #8 OR #9 OR #10 OR #11 OR #12 OR #13 OR #14 OR #15 |
| **Outcome (O):**  Economic outcomes | #17  #18  #19  #20  #21  #22  #23  #24  #25  #26  #27  #28  #29  #30  #31  #32  #33  #34  #35  #36  #37  #38  #39 | “Cost-Benefit Analysis"[Mesh]  economic analysis  economic evaluation  “cost effectiveness*”  “cost utility*”  “cost benefit*”  “ICER”  “ICUR”  "Incremental net benefit"  "INB"  "Net monetary benefit"  "NMB"  “incremental cost”  “delta cost”  “incremental effectiveness”  “incremental efficacy”  “delta effectiveness”  "Disability adjusted life years"  "DALY"  "Quality adjusted life years"  "QALY"  “monte carlo*”  "probabilistic sensitivity analysis" |
|  | #40 | Search #17 OR #18 OR #19 OR #20 OR #21 OR #22 OR #23 OR #24 OR #25 OR #26 OR # 27 OR #28 OR #29 OR #30 OR #31 OR #32 OR #33 OR #34 OR #35 OR #36 OR #37 OR #38 OR #39 |
| **I/C AND O** | #41 | Search #16 AND #40 |

**Search strategy in the National Health Service Economic Evaluation Database (NHS EED).**

| **Domain(s)** | **Search** | **Query** |
| --- | --- | --- |
| **Intervention/ Comparison:**  Among phosphate binders | #1  #2  #3  #4  #5  #6  #7  #8  #9  #10  #11  #12  #13  #14  #15 | phosphate bind*  phosphate lowering  “calcium acetate”  “calcium carbonate”  “lanthanum”  “sevelamer”  “ferric citrate”  “sucroferric”  “aluminum hydroxide”  “colestimide”  “cholebine”  “bixalomer”  “nicotinic acid”  “niacin”  “vitamin B3” |
|  | #16 | #1 OR #2 OR #3 OR #4 OR #5 OR #6 OR #7 OR #8 OR #9 OR #10 OR #11 OR #12 OR #13 OR #14 OR #15 |

**Search strategy in Cost-Effectiveness Analysis (CEA) Registry.**

| **Domain(s)** | **Search** | **Query** |
| --- | --- | --- |
| **Intervention/ Comparison (I/C):**  Among phosphate binders | #1  #2  #3  #4  #5  #6  #7  #8  #9  #10  #11  #12  #13  #14  #15 | phosphate bind*  phosphate lowering  “calcium acetate”  “calcium carbonate”  “lanthanum”  “sevelamer”  “ferric citrate”  “sucroferric”  “aluminum hydroxide”  “colestimide”  “cholebine”  “bixalomer”  “nicotinic acid”  “niacin”  “vitamin B3” |
|  | #16 | #1 OR #2 OR #3 OR #4 OR #5 OR #6 OR #7 OR #8 OR #9 OR #10 OR #11 OR #12 OR #13 OR #14 OR #15 |

**Methods S2. Data extraction from**

**Systematic Review and Meta-Analysis of the economic evaluations of Phosphate binders for treating hyperphosphatemia in Chronic Kidney Disease patients**

**Part I: General article information**

| 1. Date of data extraction | **/ / (**DD/MM/20YY) |
| --- | --- |
| 1. Study ID |  |
| 1. Reviewer | 1. K.C. 2. N.R. 3. T.S. |
| 1. First author | _______________________________________________ |
| 1. Corresponding author | _______________________________________________ |
| 1. Corresponding author’s email | _______________________________________________ |
| 1. Journal | _______________________________________________ |
| 1. Year of publication | (YYYY A.D.) |

**Part II: General study characteristics**

| 1. Country | _______________________________________________ |
| --- | --- |
| 1. Level of income country | 1. High 2. Upper- middle 3. Lower-middle 4. Low |
| 1. WHO region | 1. AFR 2. AMR 3. EMR 4. EUR 5. SEAR 6.WPR |
| 1. Type of economic evaluation | 1. CUA 2. CEA 3. CBA |
| 1. Analytic approach | 1. Study- alongside economic evaluations  1.1 Cross sectional study 1.2 Non- RCTs 1.3 RCTs  2. Model based economic evaluations  2.1 Markov 2.2 Decision tree  2.3 Discrete Event Simulation 2.4 Other____________ |
| 1. Study perspectives | 1. Societal 2. Third party payer |
|  | 3. Patient 4. Others_______________ |
| 1. Setting | 1. Country level 2. University hospital |
|  | 3. Tertiary hospital 4. Others ______________ |
| 1. Funding | 1. Yes 2. No 3. Not mentioned |
| 1. Conflicts of interest | 1. Yes 2. No 3. Not mentioned |

**Part III: General characteristics of participants and intervention/comparison**

| 1. Study participants | 1. Non-dialysis CKD with hyperphosphatemia  1.1 Stage 3 1.2 Stage 4  1.3 ESRD 1.4 Others___________  2. Dialysis CKD with hyperphosphatemia  2.1 PD 2.2 HD 2.3 not specified |
| --- | --- |
| 1. Serum phosphate level (mg/dl) | ______________ |
| 1. Serum calcium level (mg/dl) | ______________ |
| 1. Hyperparathyroidism | 1. Yes; specific serum calcium level (mg/dl) ___________  2. No 3. Not mentioned |
| 1. Sample size (n) | (For study- alongside EEs) |
| 1. Mean age (years) | . |
| 1. Intervention/ Comparison | |
| 1. Calcium catbonate; dose per days (mg/day)_________________ | |
| 2. Calcium acetate; dose per days (mg/day)_________________ | |
| 3. Any Ca- based phosphate binders (not specific) | |
| 4. Sevelamer; dose per days (mg/day)_________________ | |
| 5. Lanthanum; dose per days (mg/day)_________________ | |
| 6. Ferric citrate; dose per days (mg/day)_________________ | |
| 7. Sucroferric oxyhydroxide; dose per days (mg/day)_________________ | |
| 8. Aluminum hydroxide; dose per days (mg/day)_________________ | |
| 9. Colestimide; dose per days (mg/day)_________________ | |
| 10. Ferric citrate; dose per days (mg/day)_________________ | |
| 11.Nicotinic acid; dose per days (mg/day)_________________ | |
| 12. Any Non- Ca- based PBs (not specific) | |

**Part IV: Study methods and outcomes of economic evaluation**

| 1. Time horizon | | | 1. Lifetime  2. Other (please specify years ) | |
| --- | --- | --- | --- | --- |
| 1. Cycle length | | | Months | |
| 1. Discount rate | | | 1. Yes 2. No | |
| - 1. Discount rate for costs | | | . % | |
| - 1. Discount rate for outcomes | | | . % | |
| 1. Inflation rate | | | 1. Yes 2. No | |
| 1. Reference year of analysis | | | (YYYY A.D.) | |
| 1. Currency | | | ___________________________________________ | |
| 1. Category of costs | | | 1. Direct medical costs  2. Direct non- medical costs  3. Indirect costs  4. Not given | |
| 1. Data source of direct medical costs/ resource uses | | | 1. Clinical trials 2. Administrative data  3. Clinical databases 4. Medical records  5. Published literature 6. Others______________ | |
| 1. Data source of direct non-medical costs/ resource uses | | | 1. Clinical trials 2. Administrative data  3. Clinical databases 4. Medical records  5. Published literature 6. Others______________ | |
| 1. Data source of indirect costs/ resource usage | | | 1. Clinical trials 2. Administrative data  3. Clinical databases 4. Medical records  5. Published literature 6. Others______________ | |
| 1. Type of outcome measures | | | 1. Monetary value 2. Life years  3. QALYs 4. DALYs | |
| 1. Data source of clinical effectiveness | | | 1. Elicited in the study  2. Publised literature  2.1 Single study-based estimates; specific_______  2.2 Multiple study-based estimates  2.3. Synthesis-based estimates | |
| 1. Data source of utility | | | 1. Elicited in the study  2. Publised literature  2.1 Single study-based estimates  2.2 Multiple study-based estimates  2.3 Synthesis-based estimates  3. NA | |
| 1. Analyses of uncertainty | | | 1. One-way sensitivity analysis  2. Two-way sensitivity analysis  3. Probabilistic sensitivity analysis  4. Others____________________ 5. Not done | |
| 1. Type of cost effectiveness threshold used | | | 1. GDP per capita  1 time 2 times 3 times 4. Others____  2. Specific C/E threshold (specified method)_________ | |
| 1. C/E threshold (in currency) | | | _____________________ | |
| 1. Reported outcome | | |  | |
| 1. Costs | 1.1 with SD 1.2 with SE 1.3 with 95%Cl  1.4 with others____________ 1.5 without dispersion | | | |
| 2. Incremental costs | 1.1 with SD 1.2 with SE 1.3 with 95%Cl  1.4 with others____________ 1.5 without dispersion | | | |
| 3. Life years | 1.1 with SD 1.2 with SE 1.3 with 95%Cl  1.4 with others____________ 1.5 without dispersion | | | |
| 4. Incremental LYs | 1.1 with SD 1.2 with SE 1.3 with 95%Cl  1.4 with others____________ 1.5 without dispersion | | | |
| 5. QALYs | 1.1 with SD 1.2 with SE 1.3 with 95%Cl  1.4 with others____________ 1.5 without dispersion | | | |
| 6. Incremental QALYs | 1.1 with SD 1.2 with SE 1.3 with 95%Cl  1.4 with others____________ 1.5 without dispersion | | | |
| 7. DALYs | 1.1 with SD 1.2 with SE 1.3 with 95%Cl  1.4 with others____________ 1.5 without dispersion | | | |
| 8. Incremental DALYs | 1.1 with SD 1.2 with SE 1.3 with 95%Cl  1.4 with others____________ 1.5 without dispersion | | | |
| 9. ICER |  | | | |
| 9.1 Cost per LYs gained | | 1.1 with SD 1.2 with SE 1.3 with 95%Cl  1.4 with others____________ 1.5 without dispersion | | |
| 9.2 Cost per QALYs gained | | 1.1 with SD 1.2 with SE 1.3 with 95%Cl  1.4 with others____________ 1.5 without dispersion | | |
| 10. Cost effectiveness plane | | | | 10.1 Yes 10.2 No |
| 1. Scernario of economic parameters | | | 1 2 3 4 5 | |

**Part V: Data for pooling**

| **Study comparison** | **Intervention** | **Comparison** | **Results (Dominate/Dominant)** |
| --- | --- | --- | --- |
| I |  |  |  |
| II |  |  |  |
| III |  |  |  |

| **Base case analysis; I** | **Intervention** | **Comparison** | **Note/ comment** |
| --- | --- | --- | --- |
| Costs |  |  |  |
| Life years |  |  |  |
| QALYs |  |  |  |
| DALYs |  |  |  |
| Incremental cost |  |  |  |
| Incremental Lys |  |  |  |
| Incremental QALYs |  |  |  |
| Incremental DALYs |  |  |  |
| Incremental cost per LYs gained |  |  |  |
| Incremental cost per QALYs gained |  |  |  |
| Incremental cost per DALYs |  |  |  |
| Other |  |  |  |
| Other |  |  |  |
| Other |  |  |  |
|  |  |  |  |
|  |  |  |  |
| **Sensitivity analysis; I** | **Intervention**  **Mean ± SD**  **(95% Cl)** | **Comparison**  **Mean ± SD**  **(95% Cl)** | **Note/ comment** |
| Costs |  |  |  |
| Life years |  |  |  |
| QALYs |  |  |  |
| DALYs |  |  |  |
| Incremental cost |  |  |  |
| Incremental Lys |  |  |  |
| Incremental QALYs |  |  |  |
| Incremental DALYs |  |  |  |
| Incremental cost per LYs gained |  |  |  |
| Incremental cost per QALYs gained |  |  |  |
| Incremental cost per DALYs |  |  |  |
| Other |  |  |  |
| Other |  |  |  |
| Other |  |  |  |
|  |  |  |  |
|  |  |  |  |
|  |  |  |  |

| **Base case analysis; II** | **Intervention** | **Comparison** | **Note/ comment** |
| --- | --- | --- | --- |
| Costs |  |  |  |
| Life years |  |  |  |
| QALYs |  |  |  |
| DALYs |  |  |  |
| Incremental cost |  |  |  |
| Incremental Lys |  |  |  |
| Incremental QALYs |  |  |  |
| Incremental DALYs |  |  |  |
| Incremental cost per LYs gained |  |  |  |
| Incremental cost per QALYs gained |  |  |  |
| Incremental cost per DALYs |  |  |  |
| Other |  |  |  |
| Other |  |  |  |
|  |  |  |  |
|  |  |  |  |
| **Sensitivity analysis; II** | **Intervention**  **Mean ± SD**  **(95% Cl)** | **Comparison**  **Mean ± SD**  **(95% Cl)** | **Note/ comment** |
| Costs |  |  |  |
| Life years |  |  |  |
| QALYs |  |  |  |
| DALYs |  |  |  |
| Incremental cost |  |  |  |
| Incremental Lys |  |  |  |
| Incremental QALYs |  |  |  |
| Incremental DALYs |  |  |  |
| Incremental cost per LYs gained |  |  |  |
| Incremental cost per QALYs gained |  |  |  |
| Incremental cost per DALYs |  |  |  |
| Other |  |  |  |
| Other |  |  |  |
|  |  |  |  |
|  |  |  |  |
|  |  |  |  |

**Methods S3. ECOBIAS checklist**

| **Type of bias** | **Issues addressed (questions to consider)** | Yes/No/ Partly/ Unclear/NA |
| --- | --- | --- |
| **PART A. Overall checklist for bias in economic evaluation** | | |
| 1. Narrow perspective bias | Was a societal perspective adopted? If not, has a different perspective been justified? |  |
| 1. Inefficient comparator bias^a^ | Was the best alternative chosen as comparator?  Was current practice chosen as a comparator?  Have all comparators been described in sufficient detail? |  |
| 1. Cost measurement omission bias | Were all costs relevant to the disease and intervention identified and considered? |  |
| 1. Intermittent data collection bias | Was the resource use measured continuously? |  |
| 1. Invalid valuation bias | Is the price calculation presented in a detailed manner?  Have reference prices been used? |  |
| 1. Ordinal ICER bias | Have cardinal scales for the outcomes measure in a CEA been used? |  |
| 1. Double-counting bias | Are variables adequately checked for double-counting? |  |
| 1. Inappropriate discounting bias | Have discounting rates from guidelines been applied? |  |
| 1. Limited sensitivity analysis bias^b^ | Have the four principles of uncertainty (methodological, structural, heterogeneity, parameter) been considered in sufficient detail? |  |
| 1. Sponsor bias | Have sponsorships been disclosed? Is the study protocol freely accessible? |  |
| 1. Reporting and dissemination bias | The study/trial been listed in a trial register?  Have all results been reported according to the study protocol? |  |
| **PART B. Model-specific aspects of bias in economic evaluation** | | |
| *I: Bias related to structure* | | |
| 1. Structural assumptions bias | Is the model structure in line with coherent theory?  Do treatment pathways reflect the nature of disease? |  |
| 1. No treatment comparator bias^a^ | Is there an adequate comparator, i.e. care as usual? |  |
| 1. Wrong model bias | Is the model chosen adequate regarding the decision problem? |  |
| 1. Limited time horizon bias | Was a lifetime horizon chosen?  Were shorter time horizons adequately justified? |  |
| *II: Bias related to data* | | |
| 1. Bias related to data identification | Are the methods of data identification transparent?  Are all choices justified adequately?  Do the input parameters come from high quality and well-designed studies? |  |
| 1. Bias related to baseline data | Are probabilities, for example, based on natural history data?  Is transformation of rates into transition probabilities done accurately? |  |
| 1. Bias related to treatment effects | Are relative treatment effects synthesised using appropriate meta analytic techniques? Are extrapolations documented and well justified?  Are alternative assumptions explored regarding extrapolation? |  |
| 1. Bias related to quality of-life weights (utilities) | Are the utilities incorporated appropriate for the specific decision problem? |  |
| 1. Non-transparent data incorporation bias | Is the process of data incorporation transparent?  Are all data and their sources described in detail? |  |
| 1. Limited scope bias^b^ | Have the four principles of uncertainty (methodological, structural, heterogeneity, parameter) been considered? |  |
| *III: Bias related to consistency* | | |
| 1. Bias related to internal consistency | Has internal consistency in terms of mathematical logic been evaluated? |  |

^a^ These biases are overlapping regarding their content.

^b^ These biases are overlapping regarding their content.

**Methods S4: Data preparation and statistical analysis**

| 1. ***Data preparation***   The INB and its variance for studies included were based on the following scenarios.  *Scenario 1:* Studies that reported means along with the variance of all parameters; C and E for each treatment, ΔC, ΔE, ICER and also K. The INB and its variance were estimated by the following equation:  $INB =\Delta E (K- \mathrm{ICER})$  $\mathrm{Var}\left( \mathrm{INB} \right)\cong K^{2}\sigma_{\Delta E}^{2}+ \sigma_{ICER}^{2}$  Where $\sigma_{\Delta E}^{2}$is the variance of $\Delta E$ and $\sigma_{ICER}^{2}$ is the variance of ICER.  *Scenario 2:* Studies that reported only 95%CI of C or ΔC, E or ΔE, and ICER. The SE of ICER was estimated by following formula:  $UL= \mu+1.96 SE$  $SE= \frac{UL- \mu}{1.96}$  The INB and its variance were estimated as following equation.  $INB =\Delta E (K- \mathrm{ICER})$  $\mathrm{Var}\left( \mathrm{INB} \right)\cong K^{2}\sigma_{\Delta E}^{2}+ \sigma_{ICER}^{2}$  *Scenario 3:* Studies that reported mean and 95% CI for C or ΔC, E or ΔE, but did not report the ICER and its variance. The data of ΔC and ΔE were simulated by a Monte Carlo- simulation with 1000 simulations using gamma distribution for ΔC and normal distribution for ΔE. The ICER and covariance between ΔC and ΔE ${(\rho}_{CE}\sigma_{\Delta C}\sigma_{\Delta E})$ was estimated. As a result, the INB and its variance were estimated by the following equations.  $INB =K\left( \Delta E \right)- \Delta C$  $\mathrm{Var}\left( \mathrm{INB} \right)=K^{2}\sigma_{\Delta E}^{2}+ \sigma_{\Delta C}^{2}-2K{(\rho}_{CE}\sigma_{\Delta C}\sigma_{\Delta E})$ |
| --- |

| *Scenario 4:* Studies that reported only the mean outcome without its dispersion, but provided a cost-effective plane graph for ΔC and ΔE. Data of ΔC and ΔE were extracted from the graph using Web-Plot Digitizer software version 4.2. Subsequently, means and SE of ΔC and ΔE as well as the covariance of ΔC and ΔE, were estimated. As a result, the INB and its variance were estimated by the following equations.  $INB =K\left( \Delta E \right)- \Delta C$  $\mathrm{Var}\left( \mathrm{INB} \right)=K^{2}\sigma_{\Delta E}^{2}+ \sigma_{\Delta C}^{2}-2K{(\rho}_{CE}\sigma_{\Delta C}\sigma_{\Delta E})$  *Scenario 5:* Studies that reported only the mean outcome without its dispersion and also the cost-effective plane. Data for pooling were used from other similar studies; in terms of participant, intervention, comparator, level of income country, country or gross domestic product (GDP) per capita, model inputs (e.g., type of cost/effectiveness, type of model, discount rate, time horizon, etc.).   1. ***Currency conversion***   The currency conversion formulas were calculated as follows.  *For costing data (*$\Delta C, ICER, K^{*})$*;*  $Y_{ppp\left( in current year \right)}=Y_{€\left( in base year \right) \times}R$  *For variance of costing data (*$\sigma_{\Delta C}^{2}, \sigma_{ICER}^{2}$*);*  $V_{ppp\left( in current year \right)}=V_{€\left( in base year \right) \times}R^{2}$  $Conversion factor \left( R \right)=(\frac{{CPI}_{€\left( in current year \right)}}{{CPI}_{€\left( in base year \right)}} \times\frac{1}{ppp\left( in current year \right)} )$  Where $Y_{ppp\left( in current year \right)}$ is the final monetary unit after adjusted to US$ for the current year using PPP conversion, $Y_{€\left( in base year \right)}$ is the monetary unit in study’s currency in base year of study, R is conversion factor for standardisation costing data. Only K that came from GDP based willing- ness to pay thresholds was adjusted, not for K from country- specific C/E threshold. For K from country- specific C/E threshold, it was updated and used in current value according to their country. |
| --- |

| 1. ***Statistical analysis***   *INB pooling;*  ${INB}_{p}= \frac{\sum_{i=1}^{S} w_{i}{INB}_{i}}{\sum_{i=1}^{S} w_{i}}$   - For the fixed effects model;   $w_{i}= \frac{1}{var({INB}_{i})}$   - For the random effects model;   $w_{i}^{*}= \frac{1}{var\left( {INB}_{i} \right)+ \tau^{2}}$  $\tau^{2}= \frac{Q-(S-1)}{\sum w_{i}- \frac{\sum w_{i}^{2}}{\sum w_{i}}}$  (Q=0 if Q < S-1)  Where $Var\left( {INB}_{i} \right)$ was calculated based on the scenarios mentioned above. |
| --- |

**Table S1. Risk of bias assessment based on ECOBIAS checklist**

| **Type of bias** | **Huybrechts**  **(2005)^1^** | **Brennan**  **(2007)^2^** | **Manns**  **(2007)^3^** | **Taylor**  **(2008)^4^** | **Huybrechts**  **(2009)^5^** | **Goto**  **(2011)^6^** | **Park**  **(2011)^7^** | **Vegter**  **(2011)^8^** | **Vegter**  **(2012)^9^** | **Bernard**  **(2013)^10^** | **NICE**  **(2013)^11^** | **Thompson**  **(2013)^12^** | **Ruggeri**  **(2014)^13^** |
| --- | --- | --- | --- | --- | --- | --- | --- | --- | --- | --- | --- | --- | --- |
| **PART A. Overall checklist for bias in economic evaluation** | | | | | | | | | | | | | |
| 1. Narrow perspective bias | no | no | no | no | no | no | no | no | no | no | no | no | no |
| 1. Inefficient comparator bias^a^ | no | no | no | no | no | no | no | no | no | no | no | no | no |
| 1. Cost measurement omission | no | unclear | no | partly | no | no | no | no | no | partly | no | no | no |
| 1. Intermittent data collection | no | no | no | no | no | no | no | no | no | no | no | no | no |
| 1. Invalid valuation bias | no | unclear | no | no | no | no | no | no | no | no | no | no | no |
| 1. Ordinal ICER bias | no | no | no | no | no | no | no | no | no | no | no | no | no |
| 1. Double-counting bias | no | no | no | no | no | no | no | no | no | no | no | no | no |
| 1. Inappropriate discounting | no | no | no | no | no | unclear | no | no | no | no | no | no | unclear |
| 1. Limited sensitivity analysis^b^ | no | partly | no | partly | no | no | no | no | partly | partly | partly | no | no |
| 1. Sponsor bias | no | partly | no | no | no | no | no | no | partly | partly | no | no | unclear |
| 1. Reporting and dissemination bias | NA | NA | NA | NA | NA | no | NA | NA | NA | NA | NA | NA | no |
| **PART B. Model-specific aspects of bias in economic evaluation** | | | | | | | | | | | | | |
| *I: Bias related to structure* | | | | | | | | | | | | | |
| 1. Structural assumptions | no | no | no | no | no | no | no | no | no | no | no | no | no |
| 1. No treatment comparator^a^ | no | no | no | no | no | no | no | no | no | no | no | no | no |
| 1. Wrong model bias | no | unclear | no | no | no | no | no | no | no | no | no | no | no |
| 1. Limited time horizon bias | no | no | no | partly | no | no | no | no | no | no | no | no | partly |
| *II: Bias related to data* | | | | | | | | | | | | | |
| 1. Bias related to data identification | no | no | no | partly | no | no | no | no | no | no | no | no | no |
| 1. Bias related to baseline data | no | no | no | unclear | no | no | no | no | no | no | no | no | no |
| 1. Bias related to treatment effects | no | no | no | unclear | no | no | no | no | no | no | no | no | no |
| 1. Bias related to quality of-life weights (utilities) | no | no | no | no | no | no | no | no | no | no | no | no | NA |
| 1. Non-transparent data incorporation bias | no | partly | no | no | no | no | no | no | partly | no | no | no | no |
| 1. Limited scope bias^b^ | no | partly | no | partly | no | no | no | no | no | partly | partly | no | no |
| *III: Bias related to consistency* | | | | | | | | | | | | | |
| 1. Bias related to internal consistency | no | no | no | no | no | no | no | no | no | no | no | no | no |

^a^ These biases are overlapping regarding their content.

^b^ These biases are overlapping regarding their content.

**Table S1. Risk of bias assessment based on ECOBIAS checklist (continued)**

| **Type of bias** | **Ruggeri**  **(2015)^14^** | **Gonzalez-Parra (2015)^15^** | **Gros**  **(2015)^16^** | **Gutzwiller**  **(2015)^17^** | **Panichi**  **(2015)^18^** | **Del Pino**  **(2016)^19^** | **Nguyen**  **(2016)^20^** | **Cho**  **(2017)^21^** | **Habbous**  **(2017)^22^** | **CADTH**  **(2019)^23^** | **Yang**  **(2016)^24^** | **Goh**  **(2018)^25^** | **% unbiased studies** |
| --- | --- | --- | --- | --- | --- | --- | --- | --- | --- | --- | --- | --- | --- |
| **PART A. Overall checklist for bias in economic evaluation** | | | | | | | | | | | | | |
| 1. Narrow perspective bias | no | no | no | no | no | no | no | no | no | no | no | no | 100% |
| 1. Inefficient comparator bias^a^ | no | no | no | no | no | no | no | no | no | no | no | no | 100% |
| 1. Cost measurement omission | no | no | partly | partly | partly | no | no | partly | no | no | no | no | 72% |
| 1. Intermittent data collection | no | no | no | no | unclear | no | no | no | no | no | no | no | 96% |
| 1. Invalid valuation bias | no | no | no | no | unclear | no | partly | no | no | unclear | no | no | 84% |
| 1. Ordinal ICER bias | no | no | no | no | no | no | no | no | no | no | no | no | 100% |
| 1. Double-counting bias | no | no | no | no | unclear | no | no | no | no | no | no | no | 96% |
| 1. Inappropriate discounting | unclear | no | no | no | unclear | no | no | no | no | no | no | no | 84% |
| 1. Limited sensitivity analysis ^b^ | no | no | no | no | unclear | partly | partly | no | no | unclear | no | no | 64% |
| 1. Sponsor bias | unclear | no | no | no | no | unclear | no | no | no | no | no | unclear | 72% |
| 1. Reporting and dissemination bias | no | NA | NA | NA | no | NA | NA | NA | NA | NA | NA | NA | 100% |
| **PART B. Model-specific aspects of bias in economic evaluation** | | | | | | | | | | | | | |
| *I: Bias related to structure* | | | | | | | | | | | | | |
| 1. Structural assumptions | no | no | no | no | no | no | no | no | no | no | no | no | 100% |
| 1. No treatment comparator^a^ | no | no | no | no | no | no | no | no | no | no | no | no | 100% |
| 1. Wrong model bias | no | no | no | no | no | no | no | no | no | no | no | no | 96% |
| 1. Limited time horizon bias | partly | no | no | no | no | no | no | no | no | no | no | no | 88% |
| *II: Bias related to data* | | | | | | | | | | | | | |
| 1. Bias related to data identification | no | no | no | no | partly | no | no | no | no | no | no | no | 92% |
| 1. Bias related to baseline data | no | no | no | no | partly | no | no | no | no | no | no | no | 92% |
| 1. Bias related to treatment effects | no | no | partly | no | no | no | partly | no | no | no | no | no | 88% |
| 1. Bias related to quality of-life weights (utilities) | NA | no | no | no | no | no | no | no | no | no | no | no | 100% |
| 1. Non-transparent data incorporation bias | no | no | no | no | unclear | no | no | no | no | no | no | no | 88% |
| 1. Limited scope bias^b^ | no | no | no | no | unclear | partly | partly | no | no | no | no | no | 72% |
| *III: Bias related to consistency* | | | | | | | | | | | | | |
| 1. Bias related to internal consistency | no | no | no | no | no | no | No | no | no | no | no | no | 100% |

^a^ These biases are overlapping regarding their content.

^b^ These biases are overlapping regarding their content.

**Table S2.** **Description of the ICER and INB of included studies stratified by level of country income**

| **Treatment** | **Study** | **Threshold**  **(US$)** | **Delta cost**  **(US$)** | **Delta**  **QALYs** | | **ICER**  **(US$/QALYs)** | | **INB**  **(US$)** | | **SE of INB**  **(US$)** |
| --- | --- | --- | --- | --- | --- | --- | --- | --- | --- | --- |
| ***High-income countries*** | | | | | | | | | | |
| **Sevelamer vs CBPBs** | Manns,^3^ 2007 | 62,137.5 | 34,448.8 | 0.19 | 181,855.1 | | -22,678.1 | | 40,083.5 | |
|  | Taylor,^4^ 2008 | 42,979.9 | 12,321.3 | 0.24 | 51,242.9 | | -1,983.1 | | 33,519.2 | |
|  | Bernard,^10^ 2013 | 42,979.9 | 17,574.9 | 0.44 | 39,553.7 | | 1,522.3 | | 33,519.2 | |
|  | NICE (a),^11^ 2013 | 42,979.9 | 17,137.0 | 0.12 | 141,440.0 | | -11,913.7 | | 1,815,250.1 | |
|  | Thompson,^12^ 2013 | 42,979.9 | 67,251.0 | 1.65 | 38,107.0 | | 8,040.3 | | 33,519.2 | |
|  | Del Pino,^19^ 2016 | 58,139.5 | 45,451.4 | 1.61 | 28,179.6 | | 48,235.5 | | 44,356.7 | |
|  | Nguyen,^20^ 2016 | 71,261.7 | 33,154.1 | 0.54 | 61,867.1 | | 5,034.5 | | 53,873.8 | |
|  | Cho,^21^ 2017^a^ | 40,636.0 | 15,603.4 | 1.11 | 14,088.8 | | 29,401.0 | | 61,055.5 | |
|  | Habbous (a),^22^ 2017 | 49,710.0 | 213,139.2 | 2.49 | 85,598.1 | | -89,361.2 | | 1,674,975.6 | |
|  | Habbous (c),^22^ 2017 | 49,710.0 | 258,286.0 | 2.19 | 117,938.8 | | -149,421.0 | | 1,841,853.0 | |
| **Lanthanum vs CBPBs** | Brennan,^2^ 2007 | 42,979.9 | 856.5 | 0.02 | 48,398.4 | | -97.5 | | 4,704.7 | |
|  | Goto,^6^ 2011 | 51,535.8 | 24,064.2 | 0.63 | 38,330.0 | | 8,290.8 | | 5,762.3 | |
|  | Vegter (a),^8^ 2011 | 42,979.9 | -605,167.4 | 44.10 | -13,722.6 | | 3,034.8 | | 542.6 | |
|  | Vegter (b),^8^ 2011 | 42,979.9 | 689,070.6 | 55.80 | 12,317.6 | | 2,320.7 | | 434.1 | |
|  | Vegter,^9^ 2012 | 41,425.0 | 374,282.5 | 29.30 | 12,733.3 | | 1,201,019.8 | | 186,082.1 | |
|  | NICE (b),^11^ 2013 | 42,979.9 | 15,225.8 | 0.13 | 113,625.3 | | -9,466.5 | | 1,862,028.9 | |
|  | Gros,^16^ 2015 | 46,511.6 | -5,425.8 | 0.06 | -90,780.8 | | 8,205.7 | | 2,500.9 | |
|  | Habbous (b),^22^ 2017 | 49,710.0 | 419,135.1 | 4.22 | 99,321.1 | | -209,358.8 | | 1,780,554.8 | |
|  | Habbous (d),^22^ 2017 | 49,710.0 | 706,694.3 | 3.87 | 182,608.3 | | -514,316.5 | | 1,898,888.9 | |
| **Lanthanum vs Sevelamer** | Park,^7^ 2011 | 50,000.0 | 632.4 | 0.02 | 26,798.2 | | 547.5 | | 2,723.4 | |
|  | NICE (c),^11^ 2013 | 42,979.9 | -3,552.3 | -0.11 | 32,002.2 | | 259.1 | | 2,718.9 | |
|  | Gonzalez-Parra,^15^ 2015 | 46,511.6 | 257.0 | 0.03 | 10,226.3 | | 912.1 | | 513.4 | |
| **Sucroferric** **oxyhydroxide vs Sevelamer** | Gutzwiller,^17^ 2015 | 28,653.3 | -2,931.3 | -0.01 | 195,416.5 | | 2,501.5 | | 127,336.3 | |
|  | CADTH,^23^ 2019 | 49,710.0 | 1,062.4 | 0.03 | 35,983.3 | | 405.4 | | 214,723.9 | |
| ***Upper-middle income countries*** | | | | | | | | | | |
| **Sevelamer vs CBPBs** | Yang,^24^ 2016^a^ | 46,511.6 | 41,459.9 | 2.39 | 17,343.3 | | 69,745.7 | | 52,257.6 | |
|  | Goh,^25^ 2018^a^ | 53,066.7 | 80,995.5 | 2.08 | 38,890.6 | | 29,523.7 | | 18,882.7 | |

CBPBs; calcium-based phosphate binders, ICER; incremental cost-effectiveness ratio, INB; incremental net benefit, QALYs; Quality-adjusted life years, SE; standard error, US$; United States Dollar, vs; versus

^a^Gross domestic product (GDP) based threshold

Note; all monetary units were adjusted to current year values in 2019 using the consumer price index and converted into the United States Dollar (US$) using purchasing power parity (PPP) conversion.

**Table S3. Publication bias assessments for meta-analysis**

| **Treatment** | **INBs** | | | |
| --- | --- | --- | --- | --- |
|  | **No. of studies** | $\boldsymbol{\beta}_{\boldsymbol{1}}$ | **SE** | **Egger’s test**  **(P value)** |
| *High-income countries* | | | | |
| Sevelamer vs CBPBs | 9 | 0.04 | 0.56 | 0.941 |
| Lanthanum carbonate vs CBPBs | 7 | 1.30 | 0.44 | 0.003 |
| Lanthanum vs sevelamer | 3 | -0.23 | 0.90 | 0.798 |
| Sucroferric oxyhydroxide vs Sevelamer | 2 | -0.02 | 2.86 | 0.993 |
| *Upper-middle income countries* | | | | |
| Sevelamer vs CBPBs | 2 | 1.21 | 1.67 | 0.469 |

# $\boldsymbol{\beta}_{\boldsymbol{1}}\boldsymbol{;}$coefficient of Egger’s test, CBPBs; calcium-based phosphate binders, INBs; incremental net benefits, SE; standard error

**Table S4. Evaluation of heterogeneity of INB for lanthanum carbonate versus CBPBs through covariable meta-regression adjustment**

| **Treatment** | **Adjusting covariable** | | | | | | | | | |
| --- | --- | --- | --- | --- | --- | --- | --- | --- | --- | --- |
|  | **None** | | **Type of patients**  (pre-dialysis versus dialysis) | | **Treatments**  (first- versus second-line treatment) | | **The median discount rates** | | **The median C/E threshold** | |
|  | **No. of studies** | **I^2^ (%)** | **No. of studies** | **I^2^ (%)** | **No. of studies** | **I^2^ (%)** | **No. of studies** | **I^2^ (%)** | **No. of studies** | **I^2^ (%)** |
| Lanthanum  versus  CBPBs | 7 | 83.6 | 7 | 85.1 | 7 | 85.7 | 7 | 84.1 | 7 | 84.1 |

CBPBs; calcium-based-phosphate binders, C/E threshold; cost-effectiveness threshold, INB; incremental net benefits, No.; number, %; percent

**
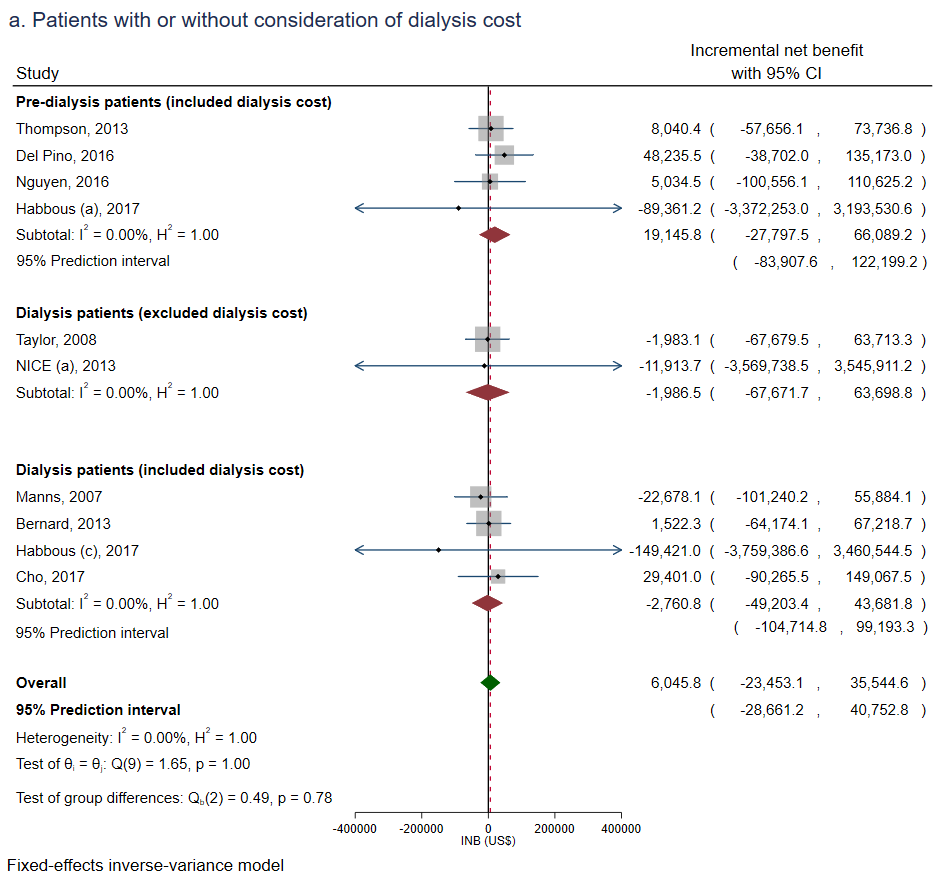
Figure S1. Subgroup analysis of pooled INBs for sevelamer versus CBPBs in high-income countries by a) patients with or without consideration of dialysis cost, and b) median cost-effectiveness (C/E) threshold.**

**
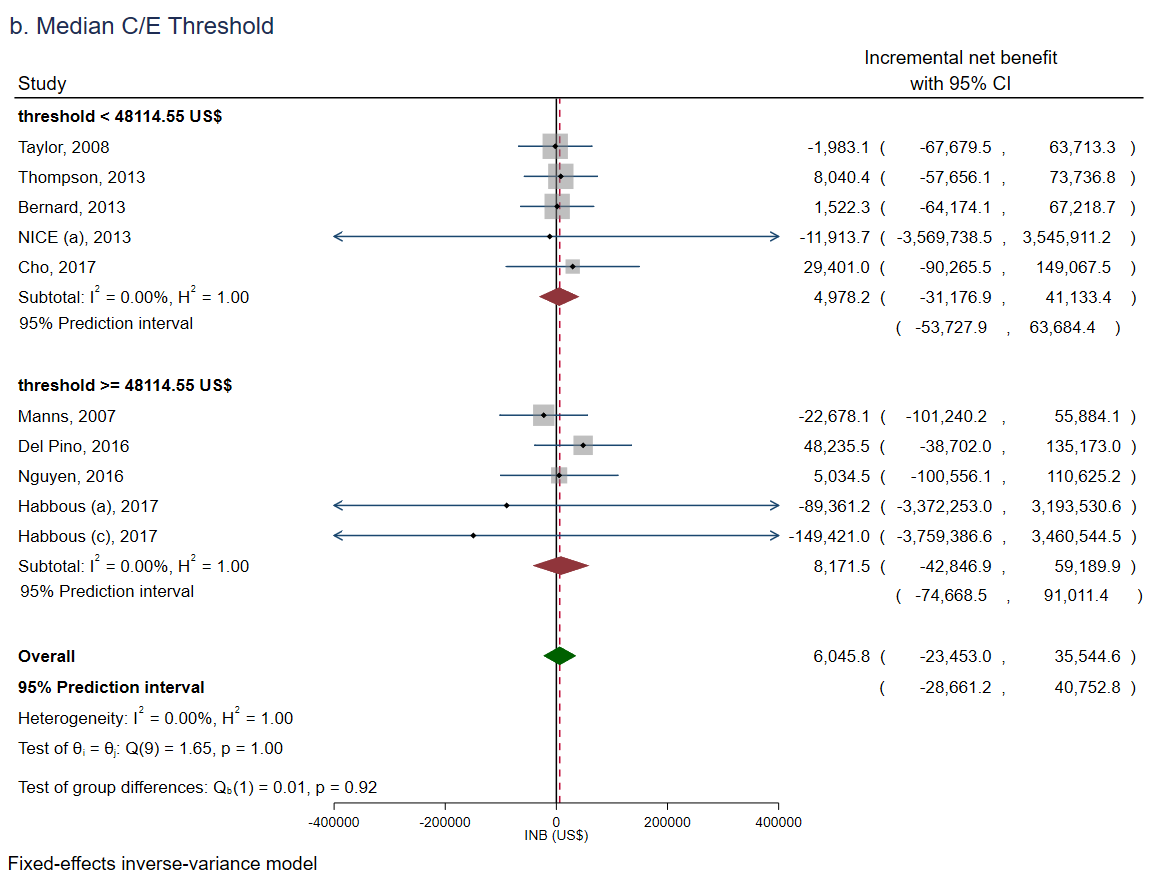
**

**Figure S2. Sensitivity analysis of pooled INBs for sevelamer versus CBPBs in high-income countries through study omission: a) non-lifetime horizon, and b) the highest threshold**

**
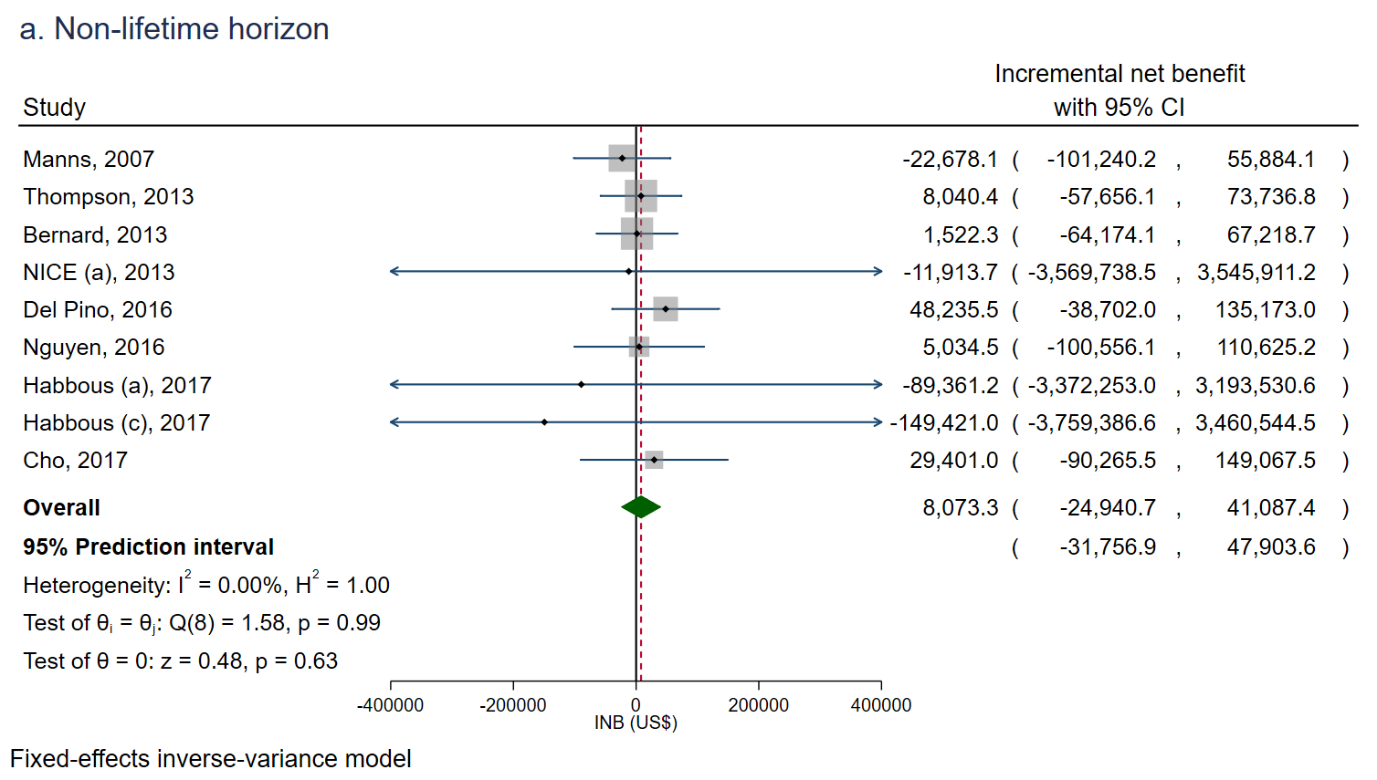
**

**
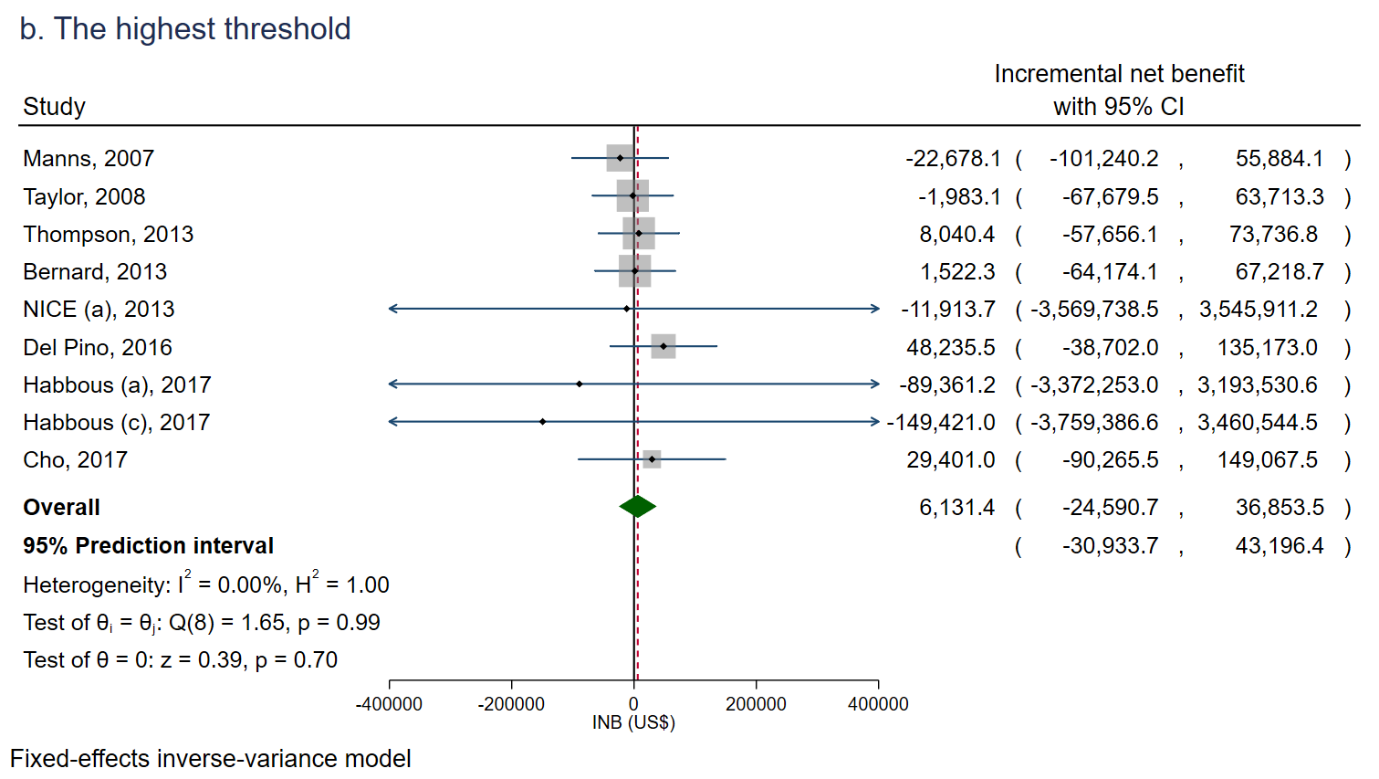
**

**Figure S3. Funnel plots for INBs of sevelamer versus CBPBs in high- and upper-middle income countries.**

**
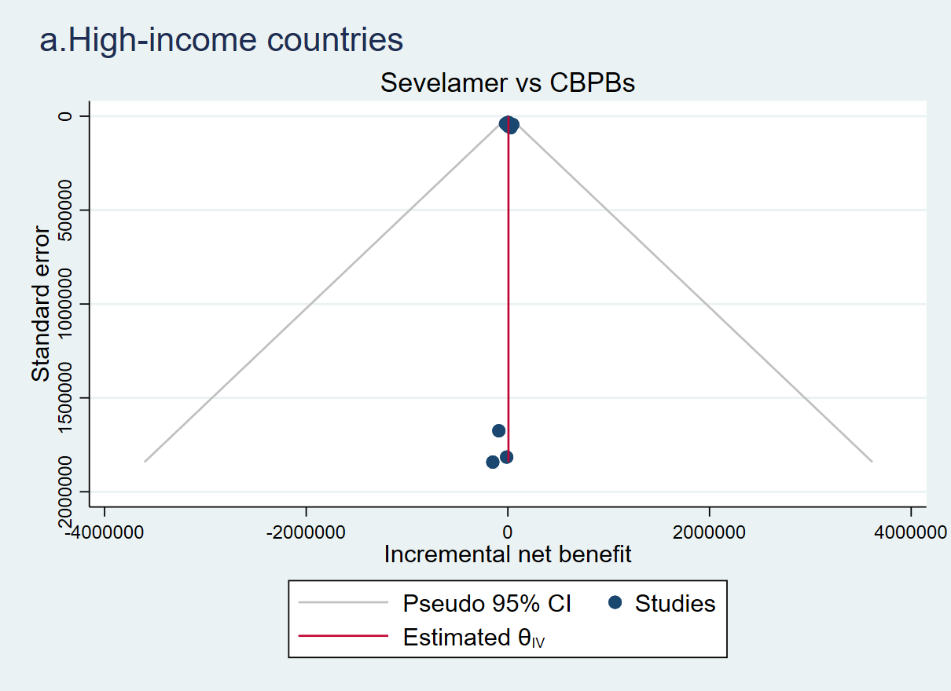
**

**
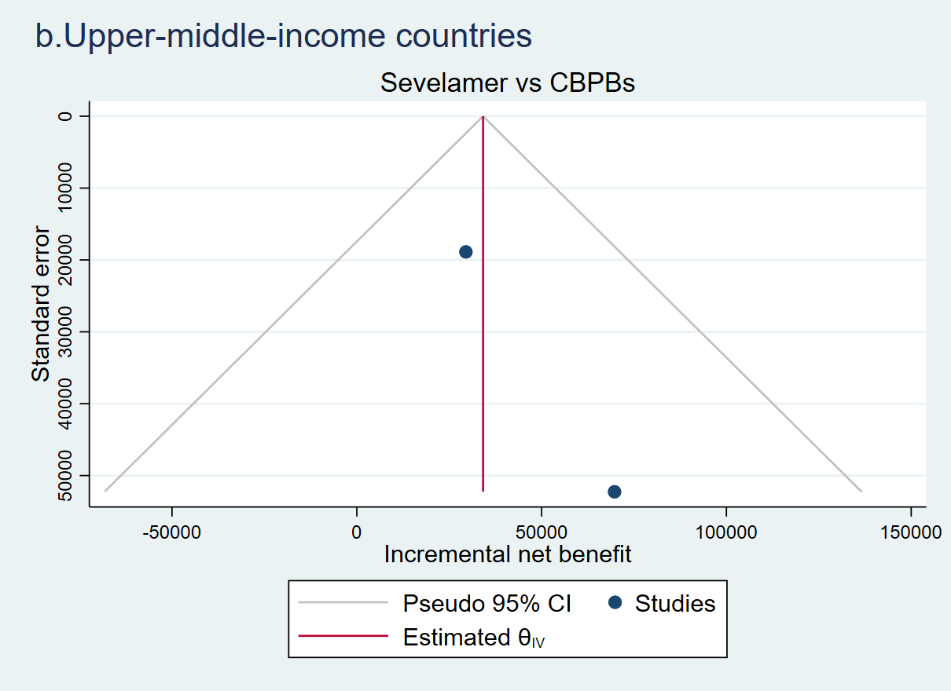
**

**Figure S4. Subgroup analysis of pooled INBs for lanthanum carbonate versus CBPBs in high-income countries by a) pre-dialysis and dialysis patients, b) treatment, c) discount rate, and d) median C/E threshold.**

**
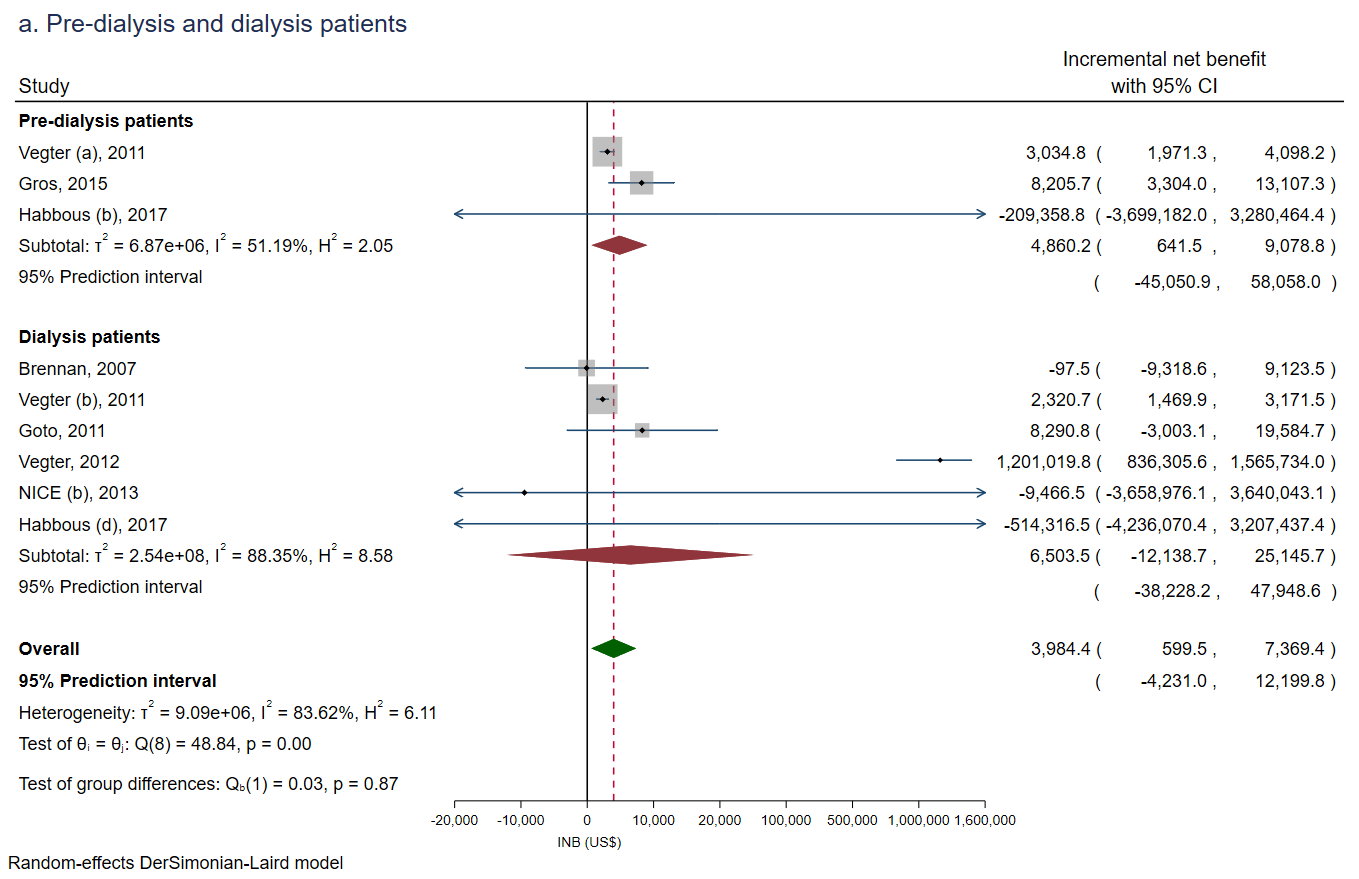
**

**
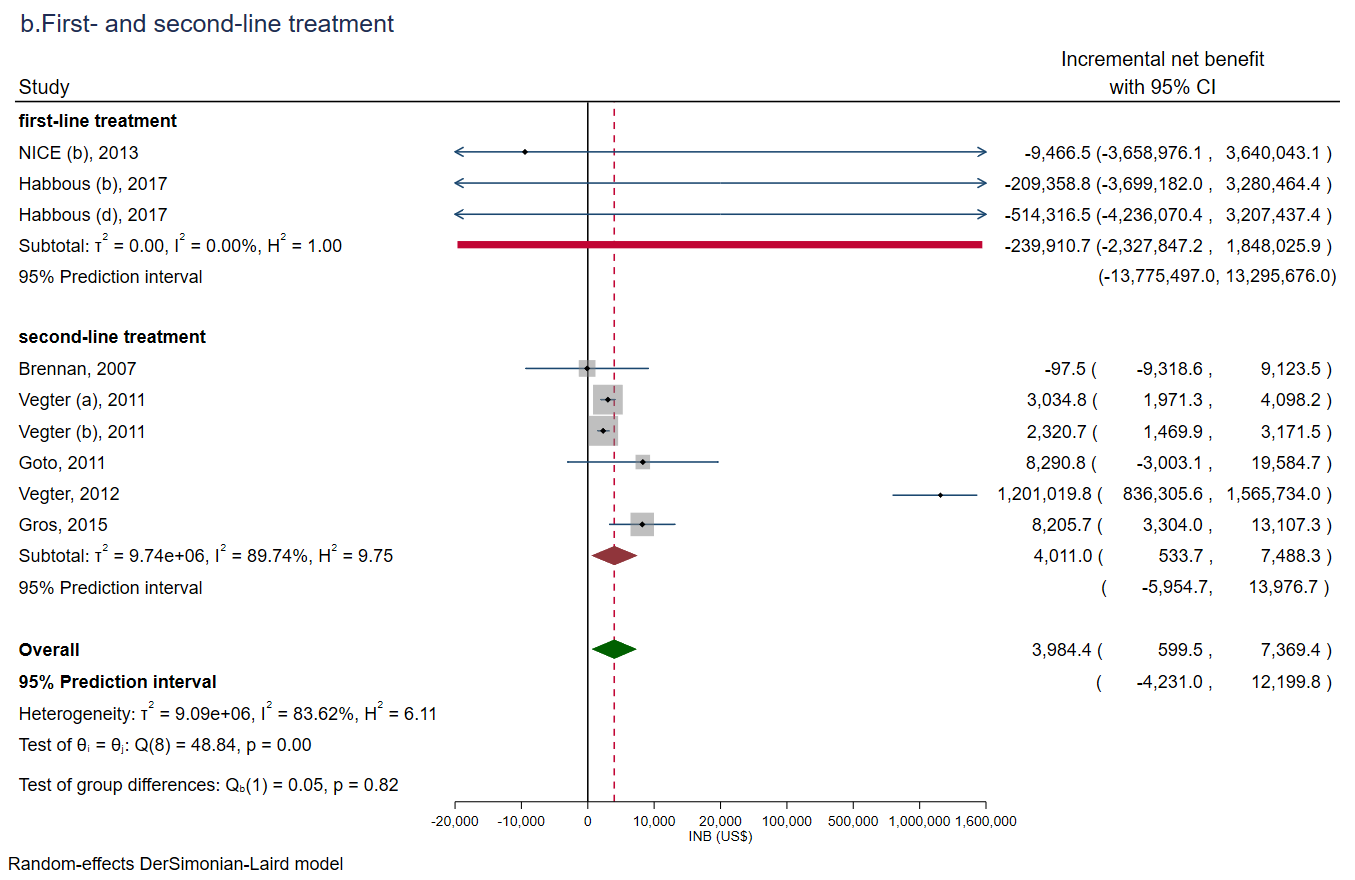
**

**Figure S4. Subgroup analysis of pooled INBs for lanthanum carbonate versus CBPBs in high-income countries by a) pre-dialysis and dialysis patients, b) treatment, c) discount rate, and d) median C/E threshold. (cont.)**

**
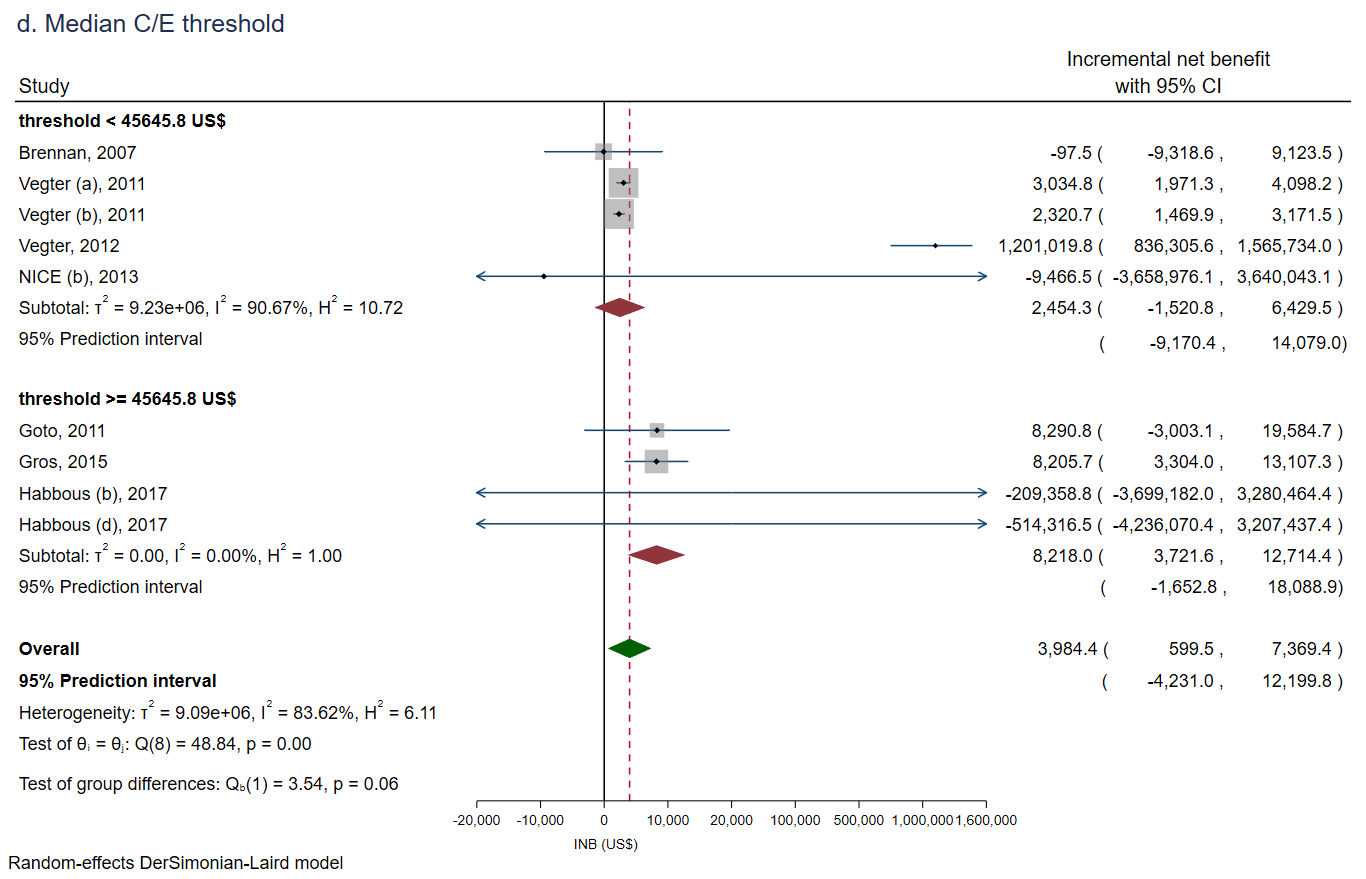

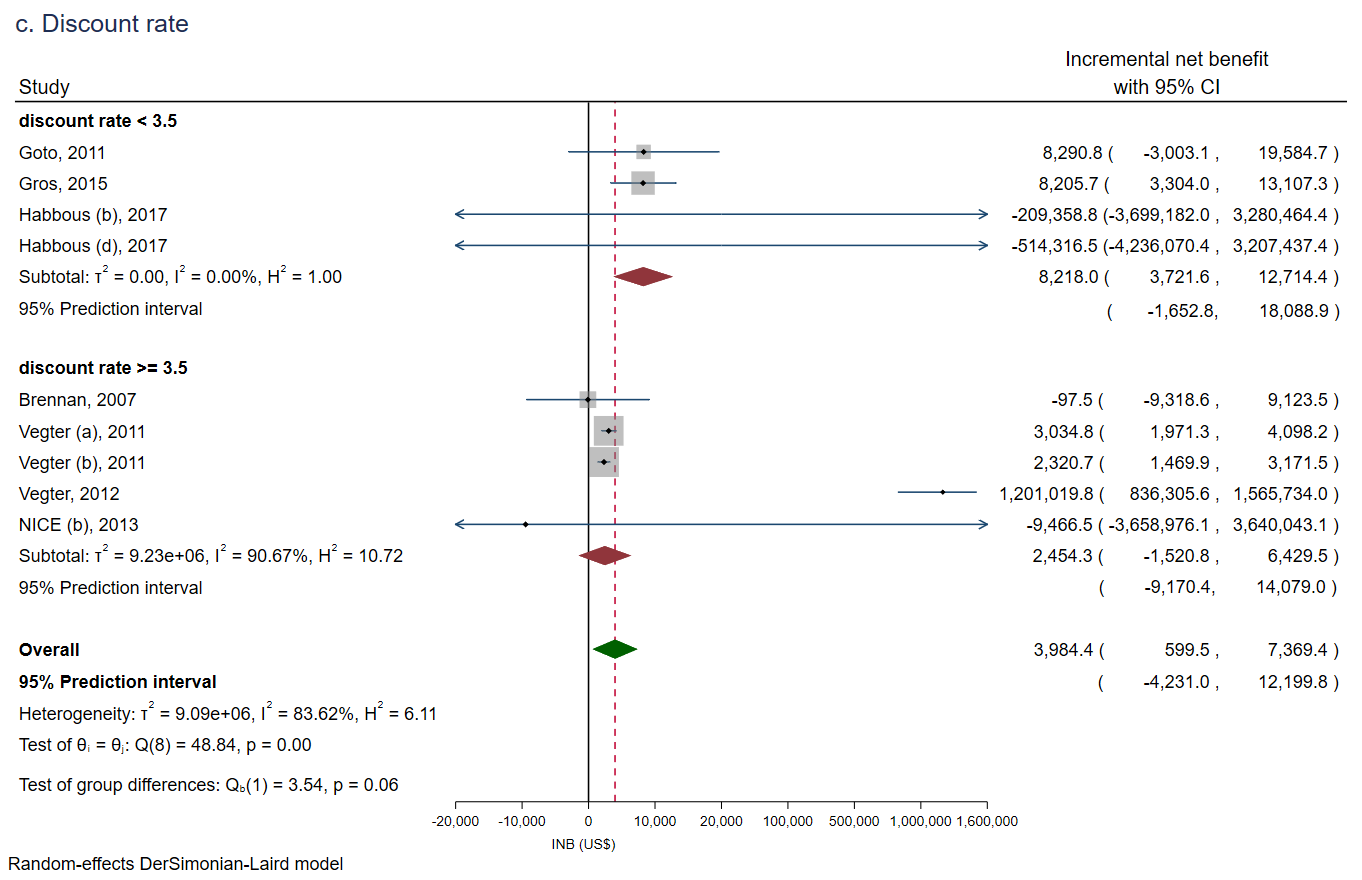
**

**Figure S5. Sensitivity analysis of pooled INBs for lanthanum carbonate versus CBPBs in high-income countries by exclusion of study: a) dialysis cost not considered, b) the highest discount rate and c) the highest C/E threshold**

**
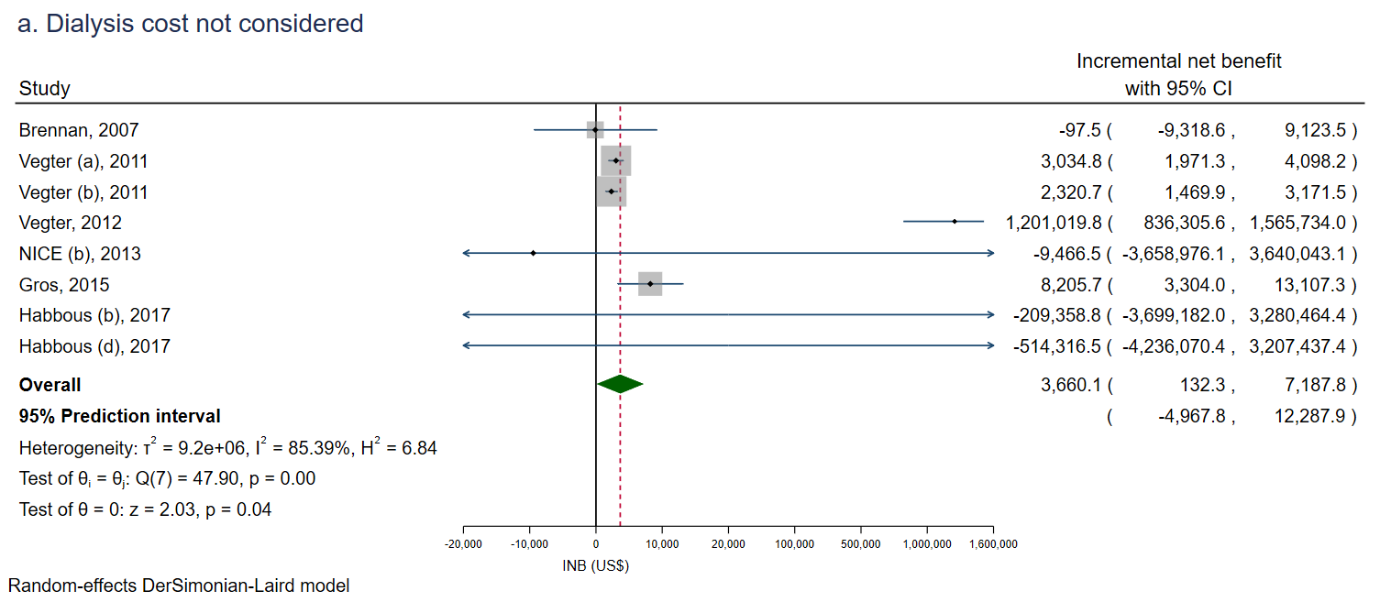
**

**
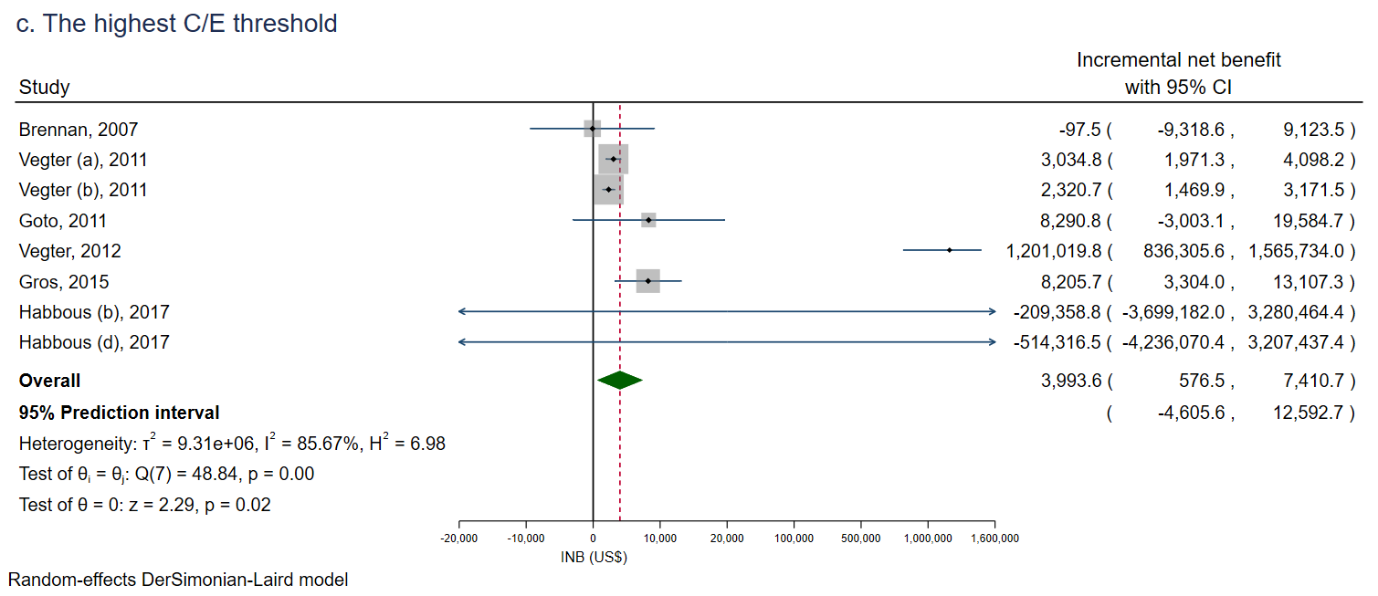

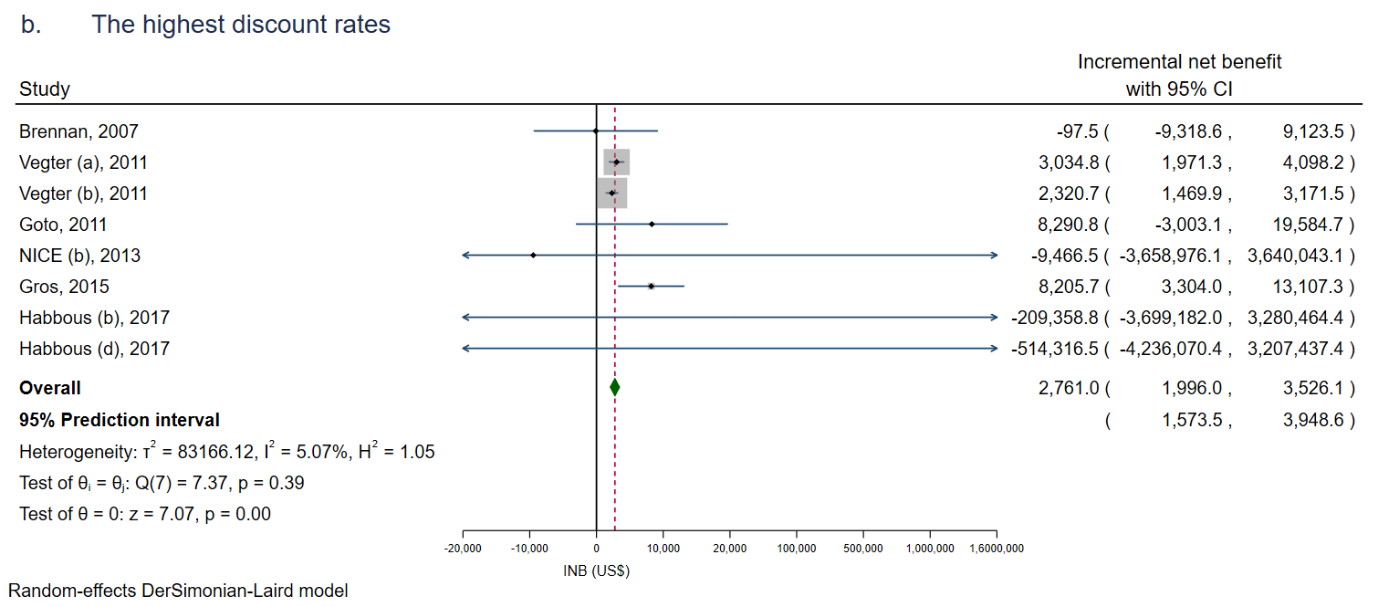
**

**Figure S6. Funnel plot and contour-enhanced funnel plot for INBs of lanthanum carbonate versus CBPBs in high-income countries**

**
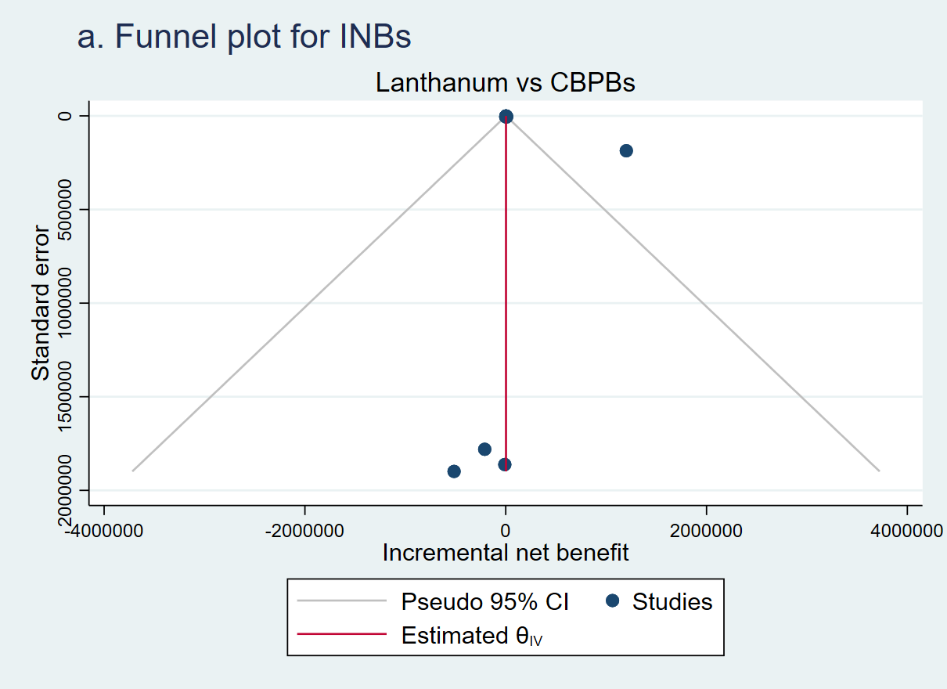
**

**
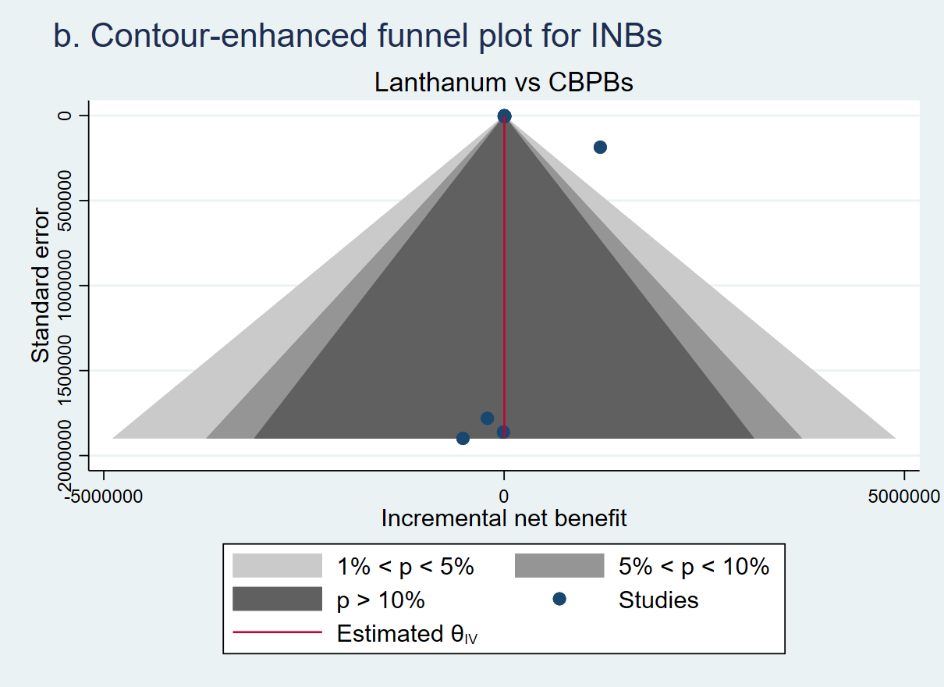
**

**Figure S7. Funnel plot for INBs of lanthanum carbonate versus sevelamer**

**
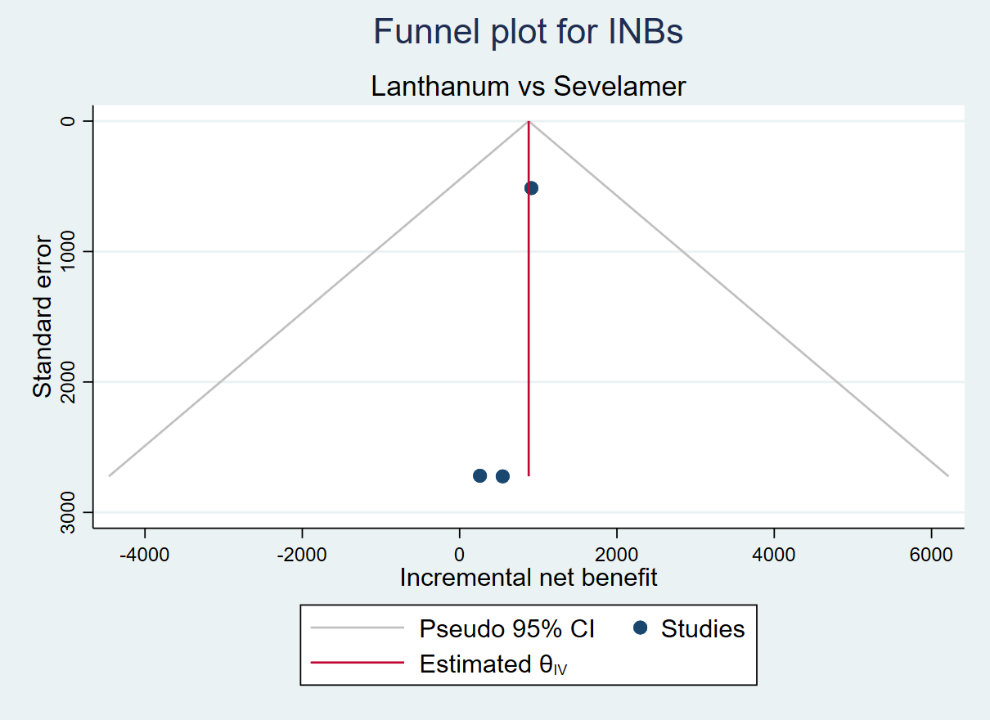
**

**Figure S8.** **Funnel plot for INBs of sucroferric oxyhydroxide versus sevelamer**

**
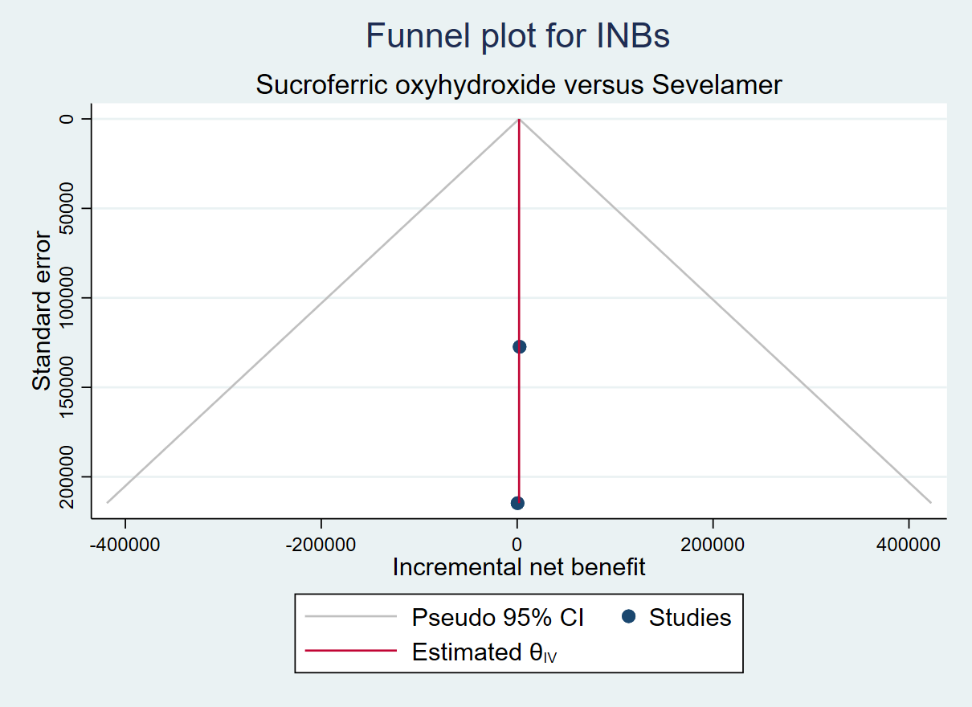
**

**REFERENCES**

1. Huybrechts KF, Caro JJ, Wilson DA, O'Brien JA. Health and economic consequences of sevelamer use for hyperphosphatemia in patients on hemodialysis. *Value in health*. 2005;8(5):549-61.

2. Brennan A, Akehurst R, Davis S, Sakai H, Abbott V. The cost-effectiveness of lanthanum carbonate in the treatment of hyperphosphatemia in patients with end-stage renal disease. *Value in health*. 2007;10(1):32-41.

3. Manns B, Klarenbach S, Lee H, et al. Economic evaluation of sevelamer in patients with end-stage renal disease. *Nephrology Dialysis Transplantation*. 2007;22(10):2867-78.

4. Taylor MJ, Elgazzar HA, Chaplin S, Goldsmith D, Molony DA. An economic evaluation of sevelamer in patients new to dialysis. *Current medical research and opinion*. 2008;24(2):601-8.

5. Huybrechts KF, Caro JJ, O'Brien JA. Prevention and management of hyperphosphatemia with sevelamer in Canada: health and economic consequences. *Value in Health*. 2009;12(1):16-9.

6. Goto S, Komaba H, Moriwaki K, et al. Clinical efficacy and cost-effectiveness of lanthanum carbonate as second-line therapy in hemodialysis patients in Japan. *Clinical Journal of the American Society of Nephrology*. 2011;6(6):1375-84.

7. Park H, Rascati KL, Keith MS, et al. Cost-effectiveness of lanthanum carbonate versus sevelamer hydrochloride for the treatment of hyperphosphatemia in patients with end-stage renal disease: a US payer perspective. *Value in Health*. 2011;14(8):1002-9.

8. Vegter S, Tolley K, Keith MS, Postma MJ. Cost-effectiveness of lanthanum carbonate in the treatment of hyperphosphatemia in chronic kidney disease before and during dialysis. *Value in Health*. 2011;14(6):852-8.

9. Vegter S, Tolley K, Keith MS, et al. Cost-effectiveness of lanthanum carbonate in the treatment of hyperphosphatemia in dialysis patients: a Canadian payer perspective. *Clinical therapeutics*. 2012;34(7):1531-43.

10. Bernard L, Mendelssohn D, Dunn E, Hutchison C, Grima DT. A modeled economic evaluation of sevelamer for treatment of hyperphosphatemia associated with chronic kidney disease among patients on dialysis in the United Kingdom. *Journal of medical economics*. 2013;16(1):1-9.

11. The National Institute for Health and Clinical Excellence (NICE). NICE clinical guideline 157 – hyperphosphataemia in chronic kidney disease (appendix F Full health economic report). London, United Kingdom: the Centre for Clinical Practice at NICE; 2009.

12. Thompson M, Bartko-Winters S, Bernard L, et al. Economic evaluation of sevelamer for the treatment of hyperphosphatemia in chronic kidney disease patients not on dialysis in the United Kingdom. *Journal of medical economics*. 2013;16(6):744-55.

13. Ruggeri M, Cipriani F, Bellasi A, et al. Sevelamer is cost-saving vs. calcium carbonate in non-dialysis-dependent CKD patients in italy: A patient-level cost-effectiveness analysis of the INDEPENDENT study. *Blood purification*. 2014;37(4):316-24.

14. Ruggeri M, Bellasi A, Cipriani F, et al. Sevelamer is cost effective versus calcium carbonate for the first-line treatment of hyperphosphatemia in new patients to hemodialysis: a patient-level economic evaluation of the INDEPENDENT-HD study. *Journal of nephrology*. 2015;28(5):593-602.

15. González-Parra E, Gros B, Galán A, et al. Análisis coste-efectividad de carbonato de lantano frente a clorhidrato de sevelámero en el tratamiento de la hiperfosfatemia en pacientes con enfermedad renal crónica en España. *PharmacoEconomics Spanish Research Articles*. 2015;12(1):11-22.

16. Gros B, Galán A, González-Parra E, et al. Cost effectiveness of lanthanum carbonate in chronic kidney disease patients in Spain before and during dialysis. *Health economics review*. 2015;5(1):14.

17. Gutzwiller FS, Pfeil AM, Ademi Z, et al. Cost effectiveness of sucroferric oxyhydroxide compared with sevelamer carbonate in the treatment of hyperphosphataemia in patients receiving dialysis, from the perspective of the National Health Service in Scotland. *Pharmacoeconomics*. 2015;33(12):1311-24.

18. Panichi V, Rosati A, Di Giorgio A, et al. A pharmacoeconomic analysis of phosphate binders cost-effectiveness in the RISCAVID study. *Blood purification*. 2015;39(1-3):174-80.

19. del Pino MD, Pons R, Rodríguez-Carmona A, Liria MR, Subirà R. Análisis coste-efectividad de sevelámero frente a carbonato cálcico en pacientes con enfermedad renal crónica no dependientes de diálisis en España. *PharmacoEconomics Spanish Research Articles*. 2016;13(2):49-56.

20. Nguyen HV, Bose S, Finkelstein E. Incremental cost-utility of sevelamer relative to calcium carbonate for treatment of hyperphosphatemia among pre-dialysis chronic kidney disease patients. *BMC nephrology*. 2016;17(1):45.

21. Cho J-H, Jang HM, Jung H-Y, et al. A real-world cost-effectiveness analysis of sevelamer versus calcium acetate in Korean dialysis patients. *Clinical therapeutics*. 2018;40(1):123-34.

22. Habbous S, Przech S, Martin J, Garg AX, Sarma S. Cost-Effectiveness of First-Line Sevelamer and Lanthanum versus Calcium-Based Binders for Hyperphosphatemia of Chronic Kidney Disease. *Value in Health*. 2017;21(3):318-25.

23. Canadian Agency for Drugs and Technologies in Health (CADTH). Pharmacoeconomic Review Report: Sucroferric Oxyhydroxide (Velphoro): (Vifor Fresenius Medical Care Renal Pharma Ltd.): Indication: For the control of serum phosphorus levels in adult patients with end-stage renal disease on dialysis. Ottawa (ON); 2019.

24. Yang L, Tan SC, Chen C, et al. Economic evaluation of sevelamer versus calcium-based binders in treating hyperphosphatemia among patients with end-stage renal disease in China. *Clinical therapeutics*. 2016;38(11):2459-67. e1.

25. Goh B, Soraya A, Goh A, Ang K. Cost-Effectiveness Analysis for the Treatment of Hyperphosphatemia in Predialysis Patients: Calcium-Based versus Noncalcium-Based Phosphate Binders. *International journal of nephrology*. 2018;2018.
